# Proximity-dependent proteomics and network analysis of adenylyl cyclase isoforms 5, 6, and 9 in cardiomyocytes

**DOI:** 10.1016/j.jbc.2025.110539

**Published:** 2025-07-31

**Authors:** Taeyeop Park, Yong Li, Neha Arora, Anibal Garza-Carbajal, Karen Colwill, Cassandra J. Wong, Yong Zhou, Carmen W. Dessauer

**Affiliations:** 1 Department of Integrative Biology and Pharmacology, McGovern Medical School at the University of Texas Health Science Center, Houston, Texas, USA; 2 Lunenfeld-Tanenbaum Research Institute, Sinai Health, Toronto, Ontario, Canada

**Keywords:** adenylyl cyclase, cyclic AMP (cAMP), cardiomyocyte, intracellular trafficking, proximity proteomics, epidermal growth factor receptor (EGFR), ryanodine receptor (RYR2), sarcoplasmic reticulum (SR), Sarco/endoplasmic reticulum Ca^2+^-ATPase 2 (SERCA2), syntrophin alpha 1 (SNTA1), vesicle-associated membrane protein 2 (VAMP2)

## Abstract

cAMP influences multiple aspects of cardiac biology, including the regulation of contraction, relaxation, and overall stress responses. The functional outcomes of cAMP are driven by the spatial arrangement of enzymes that produce and degrade cAMP (adenylyl cyclase [AC] and phosphodiesterase, respectively), together with the downstream targets of cAMP. We performed proximity-dependent biotin identification (BioID) of near-neighbor interactions proteomics in cardiomyocytes to generate proximity maps for three cardiac AC isoforms. Processing and trafficking functions were the most common gene ontology terms for ACs, with AC5 and AC6 generating unique proximal protein sets compared to AC9. AC5/6 proximal proteins showed significant localization at the endo/sarcoplasmic reticulum with roles in calcium handling, whereas those near AC9 were more abundant at the plasma membrane. Upon treatment with the hypertrophic stimulus norepinephrine, only a few calcium handling proteins were differentially labeled for AC5/6, but not AC9. Endogenous AC activity copurified with vesicle transport, signaling, and muscle contraction proteins that were identified by BioID and/or pulldown of FLAG-tagged AC (FLAG-mass spectrometry) in cardiomyocytes, including ryanodine receptor 2, ATP2A2 (sarco/endoplasmic reticulum Ca^2^^+^-ATPase 2), epidermal growth factor receptor, syntrophin alpha 1, and vesicle-associated membrane protein 2. Finally, overlapping BioID and FLAG-mass spectrometry datasets suggested heterodimerization of AC5 and AC6, while super-resolution electron microscopy spatial mapping validated homodimerization and heterodimerization of these AC isoforms on the plasma membrane. Overall, our comprehensive network analysis has identified new binding partners and shed light on the spatial and functional significance of AC-containing macromolecular complexes in heart.

The cAMP signaling pathway plays a crucial role in regulating cardiac function and adaptation to stress. It mediates the effects of the sympathetic and parasympathetic nervous systems on cardiac contractility and relaxation by modulating the activity of key proteins involved in excitation–contraction coupling, including ryanodine receptors, RAD-regulated L-type calcium channels, and phospholamban (PLN)-regulated sarcoplasmic reticulum (SR)/endoplasmic reticulum (ER) Ca^2+^-ATPase (SERCA) (1, 2, 3, 4, 5, 6). The cAMP pathway also influences cardiac growth and remodeling by affecting gene expression and protein synthesis. Abnormalities in cAMP signaling can lead to cardiac hypertrophy, arrhythmias, and heart failure. One of the key features of this pathway is the formation of cAMP nanodomains, where cAMP levels are distinct from the surrounding cytoplasm. These nanodomains are generated by the spatial and temporal coordination of cAMP synthesis by adenylyl cyclase (AC), cAMP degradation by phosphodiesterase (PDEs), and cAMP detection by effectors such as PKA, Rap guanine nucleotide-exchange proteins, and cyclic nucleotide-gated channels (7, 8). Within these nanodomains, cAMP can modulate the activity of specific targets and signalosomes, leading to diverse physiological outcomes. However, the overall complexity and dynamics of AC-containing nanodomains is largely unknown.

In cardiomyocytes (CMs), cAMP production is largely controlled by two AC isoforms, AC5 and AC6, which have similar regulatory properties but distinct phenotypes upon overexpression and deletion. For example, AC6 overexpression has a protective effect in ischemia and dilated cardiomyopathy models but not chronic pressure overload (9, 10, 11), while AC5 deletion is beneficial in models of chronic heart failure (12) (and reviewed in (13, 14, 15)). The individual roles of AC5 and AC6 may be related to their differential subcellular localization, which affects the spatial distribution of cAMP and PKA signaling. For example, AC5 is coupled to β1 and β2-adrenergic receptor (AR) stimulation of L-type calcium channel and localized mainly to transverse tubules (T-tubules), while AC6 couples largely to β1AR and resides outside the T-tubules (16, 17). Although AC9 is expressed at very low levels in heart, it has important functions in regulation of cardiac repolarization, heart rate, and stress responses (18). For example, AC9 is important for cardioprotective PKA phosphorylation of HSP20 at baseline and interacts with Yotiao, an A-kinase–anchoring protein (AKAP), to regulate the I_KS_ current and repolarization of the heart in response to sympathetic stimulation. Finally, complexes of AC and the cAMP effector, POPDC1, regulate heart rate control (19). Thus, each AC isoform drives overlapping aspects of cardiac function and stress responses (14, 18, 20).

To gain insights into the unique composition of signalosomes that surround ACs and the overall trafficking and organization of AC enzymes, we sought to map the near-neighbor protein sets for three AC isoforms in CMs. To capture weak and possibly transient interactions of ACs, we have employed a proximity-dependent biotin identification (BioID) of near-neighbor interactions approach where an abortive biotin ligase (miniTurbo [mT]) and FLAG epitope tag were fused to the C terminus of GFP and AC enzymes (AC5, AC6, and AC9) to biotin-label proteins within ∼10 nm of the biotin ligase-fused bait proteins (21, 22, 23). Fusion proteins were expressed in primary neonatal rat CMs, incubated for 3 h with biotin, and subsequently used for streptavidin pull-down and mass spectrometry (MS) analysis. We also detected protein complexes by FLAG-tag pulldown followed by MS (FLAG-MS). High-confidence near-neighbor proteins were identified by Significance Analysis of INTeractome (SAINT) and classified by their enriched gene ontology (GO) terms and cellular compartments. AC5 and AC6 generated overlapping proximal protein sets as compared to AC9, suggesting possible heterodimerization. Electron microscopy (EM) spatial mapping supported both homodimerization and heterodimerization of AC5/6. Surprisingly, chronic hypertrophic treatment with norepinephrine displayed few alterations in AC proximal proteins. Importantly, cardiac proteins with known functional roles in contractility and calcium handling segregated with individual AC isoforms. FLAG-MS was used as a complementary method to BioID, providing overlapping interactomes related to vesicle trafficking, signaling, and muscle contraction. Moreover, endogenous AC activity copurified with pulldowns of ATP2A2 (SERCA2), ryanodine receptor 2 (RYR2), epidermal growth factor receptor (EGFR), vesicle-associated membrane protein 2 (VAMP2; also known as synaptobrevin-2), and syntrophin alpha 1 (SNTA1) from neonatal CMs. Overall, our comprehensive network analysis provides insights for understanding the spatial and functional significance of AC-containing complexes in heart and supports the homodimerization and heterodimerization of AC5/6 in CMs.

## Results

### Proximity-dependent biotinylation strategy and validation of AC-fusion proteins

**Figure 1:**
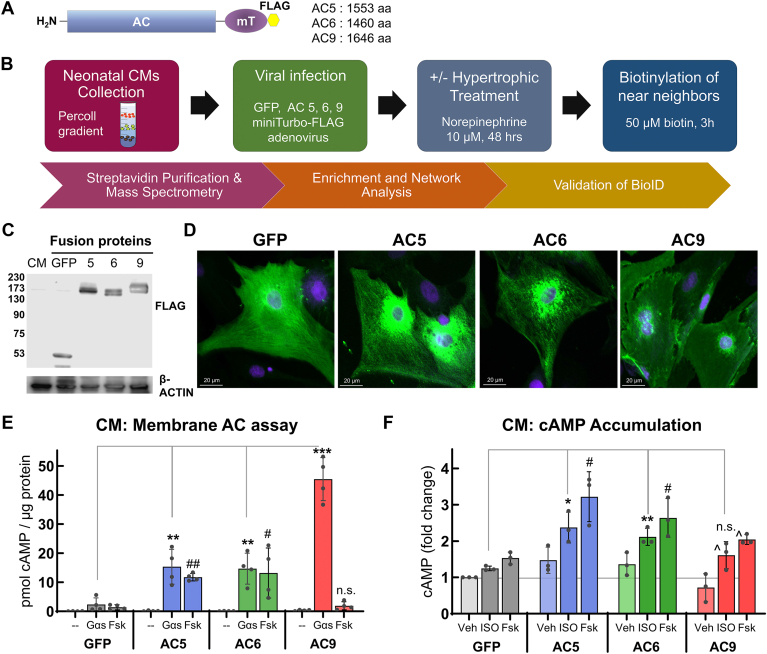
**Strategy for AC-specific BioID near-neighbor analysis.***A*, schematic of AC-miniTurbo (mT)-Flag fusion proteins. *B*, overview of BioID experimental workflow in primary neonatal CMs. Biotinylated proteins were isolated by streptavidin pulldown and identified *via* MS. Proximity data sets for each AC protein were compared against uninfected and GFP-mT control datasets (using SAINTexpress) to identify high-confidence proximity prey proteins for use in bioinformatic analysis and validation assays. *C*, Western blotting of BioID proteins expressed in CMs. Proteins in whole cell lysates were detected using FLAG antibody, with β-ACTIN as a protein loading control (n = 3). *D*, subcellular localization of BioID constructs in neonatal CMs. Confocal images of cardiomyocytes expressing GFP- or AC-mT fusion proteins. Localization of fusion proteins (anti-FLAG, *green*) and nucleus (Hoechst dye, *blue*) is shown. The scale bar represents 10 μm. *E*, AC activity of membranes isolated from neonatal CMs expressing AC-mT fusion proteins. Membranes were stimulated with 300 nM Gαs or 50 μM Fsk and cAMP levels were measured by EIA assay. Data are shown as mean ± SD, n = 4 separate experiments, performed in duplicate. Statistics: ∗, #*p* < 0.05; ∗∗, ##*p* < 0.01. Gαs∗ and Fsk# treatments were analyzed separately by one-way ANOVA with Dunnett’s multiple comparison test *versus* GFP control group. *F*, cAMP accumulation in neonatal CMs. Expression of AC5- and AC6-mT fusion proteins significantly increased isoproterenol- (ISO) and forskolin- (Fsk) stimulated cAMP accumulation compared to GFP-mT and vehicle (Veh). Data are shown as mean ± SD, n = 3 experiments, performed in duplicate. Statistics: ∗, #*p* < 0.05; ∗∗, *p* < 0.01 (as in 1E). ^, *p* < 0.05 unpaired *t* test compared to AC9 vehicle. AC, adenylyl cyclase; BioID, biotin identification; CM, cardiomyocyte; MS, mass spectrometry; mT, miniTurbo.

We employed a BioID proteomics technique to examine the near-neighbor proteins of three physiologically important AC isoforms in the heart. This technique utilizes an engineered abortive biotin protein ligase, mT, to label proteins in living cells present in close proximity to the enzyme (21, 22). We fused a FLAG-tag and the 28 kD mT to GFP (as a control) and to the C terminus of AC 5, 6, and 9 (Figs. 1*A*, and S1*A*). Each construct was virally expressed in primary neonatal CMs. The overall strategy for identification of near-neighbor proteins for individual ACs and the subsequent validation of their function is shown in Figure 1*B*. In short, we (1) purified and identified proximal labeled proteins (referred to as prey) specific to each AC isoform *via* comparison with control GFP and uninfected groups, (2) performed functional enrichment and localization analysis of high-confidence proteins, and (3) performed FLAG-MS to identify protein complexes, and (4) tested a subset of hits by coimmunoprecipitation with endogenous ACs.

To validate the functional integrity of our AC fusion proteins, we measured the expression of each construct and their biotinylation of cellular proteins by Western blot analysis and immunocytochemistry (Fig. 1, *C* and *D*). Upon incubation with biotin, protein biotinylation can be detected in as little as 1 to 3 h in CMs (Fig. S1*B*). The subcellular localization of AC-mT-FLAG fusion proteins is primarily observed in the sarcolemma and perinuclear regions, as opposed to the GFP-mT-FLAG control which is largely cytoplasmic. *In vitro* AC enzymatic assays were performed with membrane preparations from CMs expressing AC-mT or GFP-mT fusions and basal, Gαs-, and forskolin-stimulated AC activity was measured (Fig. 1*E*). AC5 and AC6 showed an ∼2- to 3-fold increase in Gαs and forskolin-stimulated activity compared to the GFP-mT control. AC9 is largely forskolin insensitive (24, 25, 26) and displayed no enhancement of forskolin-stimulated activity compared to membranes expressing GFP-mT, but displayed a 19-fold increase in Gαs-stimulated activity compared to GFP-mT (GFP: 2.20 pmole/μg and AC9: 42.29 pmole/μg). Isoproterenol treatment (10 min) increased cAMP accumulation in CMs expressing AC5-mT and AC6-mT by 1.89 ± 0.21 and 1.69 ± 0.08-fold, respectively, compared with GFP-mT (Fig. 1*F*). Similarly, forskolin responses were also enhanced in AC5- and AC6-mT expressing CMs. Isoproterenol responses were not significantly enhanced by AC9-mT compared to GFP-mT (*p* = 0.17) but were significant compared to AC9-mT vehicle (*p* = 0.044). AC9 did show a 33% increase in cellular forskolin responses compared to GFP-mT (*p* = 0.01). This is consistent with previous reports of forskolin stimulation of AC9 in the presence of Gαs (25, 26, 27). Overall, viral expression conditions were utilized that express AC 5, 6, and 9 at ∼2- to 3-fold over endogenous AC activity to enhance isoproterenol- and Gαs-stimulated activities in CMs. As a final test of functional fusion proteins, we measured the interaction of AC5-mT with several known cardiac binding proteins, namely Gαs, and Gβγ (15). Both proteins were pulled down with AC5-mT, upon short-term (2 min, 10 nM) isoproterenol treatment (Fig. S1*C*), while interactions of AC 5-, 6-, and 9-mT, but not GFP-mT, were also observed by proximity ligation assay with endogenous Gβγ (Fig. S1*D*).

### GO enrichment and subcellular localization analysis of AC near-neighbor proteins

**Figure 2:**
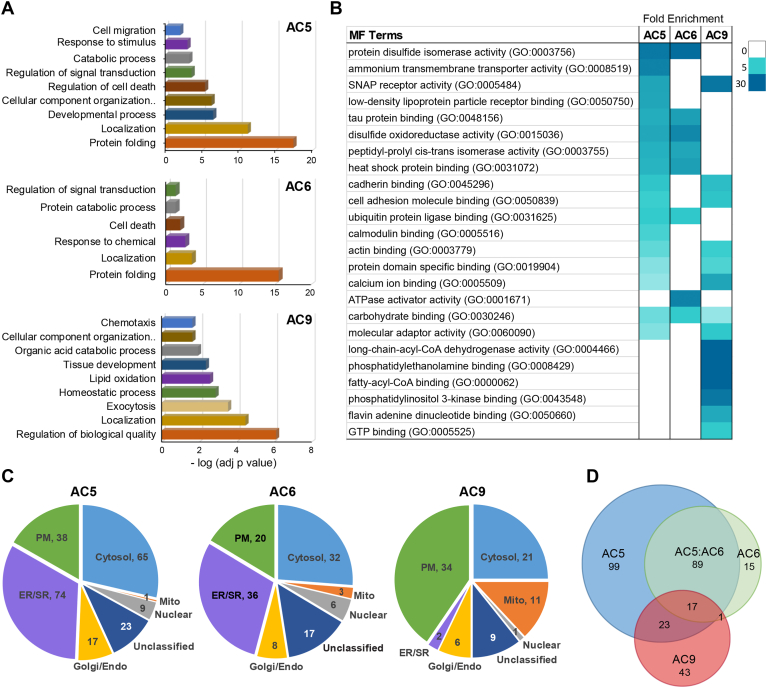
**Distribution of gene ontology enrichment and subcellular localization analysis of prey proteins.***A*, gene ontology (GO) classification of the proteins associated with specific AC isoforms in neonatal CMs. Prey proteins identified by MS analysis were submitted to gProfiler for functional enrichment analysis. The *top* nonredundant enriched biological process (BP) are displayed according to their −log adjusted *p* value. *B*, molecular function (MF) and pathway enrichment analysis of prey proteins. The fold enrichment value of highly ranked GO:MF pathways are shown, with a false discovery rate (FDR) cutoff ≥0.07 and statistical assessment by Fisher’s exact test. *C*, subcellular distribution of AC-specific prey proteins. Subcellular compartments were assigned using SubCellBarCode and UniProtKB. The number of proteins within each compartment is depicted in the Venn diagram. *D*, overlap of high-confidence proximal proteins for each AC isoform. The number of proteins in each set of the Venn diagram is shown. AC, adenylyl cyclase; CM, cardiomyocyte; MS, mass spectrometry.

To perform BioID, primary neonatal CMs were infected 2 days postisolation with adenoviruses encoding the GFP-mT and AC-mT constructs. On day 5, CMs were incubated with biotin for 3 h and harvested for streptavidin-affinity purification and subsequent liquid chromatography-tandem mass spectrometry (LC-MS/MS) (Figs. 1*B*, S1*A*, and S2). To obtain high-confidence lists, MS data were scored by SAINT, using a SAINT score (SS) cut-off of ≥0.7 (Bayesian False Discovery Rate ≤ 0.03) compared with the GFP-mT and biotin-treated control CM groups (n = 14 combined). Proteins with high confidence (192 for AC5, 83 for AC6, and 72 for AC9) were subjected to further bioinformatics analysis using GO enrichment analysis. The top statistical representative GO:biological process terms for all ACs were associated with protein folding/regulation of biological quality (GO:0006457/GO 0065008) and localization (GO:0051179) categories (Fig. 2*A* and Table S1). Within the protein folding terms were enzymes for glycosylation, a known modification of all ACs (15). Although it is certainly true that ACs and cAMP are important regulators of stress responses in CMs and heart, we cannot rule out the possibility that the protein folding-related terms could be a consequence of adenoviral expression of ACs. Therefore, we focused our analysis on nonfolding related processes.

Pathological hypertrophy is associated with alterations in G protein coupled receptor (GPCR) localization and signaling from different regions of the plasma membrane (PM) (*e.g.* T-tubules *versus* sarcolemma regions) (1, 17). To measure potential alterations in near neighbors for ACs upon CM hypertrophy, we compared vehicle *versus* 48 h norepinephrine (NE) treatment (prior to incubation with biotin). Cell size, atrial- and B-type natriuretic peptides (ANP and BNP) expression confirmed cellular hypertrophy (Fig. S3, *A* and *B*). To our surprise, only a small subset of proteins was differentially labeled for AC5 (11), AC6 (5), or AC9 (0) upon NE treatment (Fig. S3*C*). Most of the differentiated proteins proximal to AC5 were calcium-binding proteins (8 of 12), consistent with hypertrophic changes in calcium handling (28), whereas AC6 differentially labeled proteins residing in the ER/SR or intercalated disc. As few significant alterations were identified, vehicle and NE groups were combined for subsequent classification analysis.

Using the PANTHER classification system, we performed molecular function analysis of proteins labeled by each AC isoform (criteria: SS ≥ 0.7, Fig. 2*B*). Functional terms associated with protein folding and processing (GO:0003756, GO:0051787, and GO:0015036) were once again among the highest for AC5 and AC6, but absent for AC9. AC5/6 were also selectively associated with cell stress terms, including heat shock protein binding (GO:0031072) and ubiquitin protein ligase binding (GO:0031625). AC5 and AC9 were associated with functional terms related to cell–cell contacts and actin cytoskeleton (SNAP receptor activity GO:0005484, cadherin binding GO:0045296, cell adhesion GO:0050839, and actin binding GO:0003779), while all three AC isoforms were associated with terms related to scaffolding or molecular adaptor activity (GO:0060090). Transmembrane transporter activity (*i.e.* ammonium GO:0008519) for AC5 arose due to labeling of aquaporin (AQP1) and several solute carrier family genes (Slc12A4, Slc12A7, Slc33a1), important for cell volume homeostasis or small peptide cellular uptake. AC9 had one of the most surprising groups of functional terms: fatty-acyl-CoA binding (GO:0000062) and phosphatidylethanolamine binding (GO:0008429), associated with lipid modification (GO:0005543 and GO:0008289) (Fig. 2*B*). Finally, SubCellBarCode and UniProtKB were used to classify subcellular compartments for proximal proteins (SS ≥ 0.7), which included localization to the cytosol (25–29%), ER/SR (33%, 30%, and 2% in AC5, AC6, AC9, respectively), PM (17% for AC5/6 and 41% for AC9), and Golgi/endosomes (∼7%) (Fig. 2*C*). While direct immunofluorescence of endogenous ACs remains challenging due to technical limitations of AC antibodies, near-neighbor proteins of ACs appeared widely distributed in many organelles, with AC5/6 proximal to considerably more ER/SR localized proteins than AC9. These subcellular differences are consistent with the strong 86% overlap of AC6 proximal proteins with those of AC5, while AC9 showed only a 48% and 21% overlap with AC5 and AC6 proximal proteins, respectively (Fig. 2*D*).

**Figure 3:**
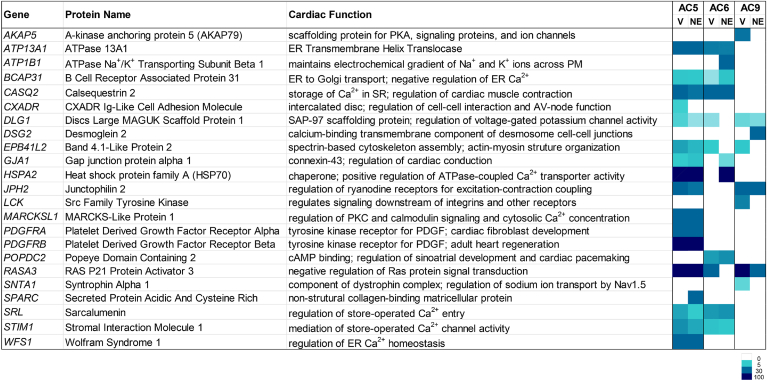
**Cardiac function analysis for AC isoforms.** Prey proteins (SS ≥ 0.7) included in GO terms for heart contraction (GO:0008016) and Ca^2+^ ion transport (GO:0051924) are shown based on fold change over GFP control. Cardiac biological process and phenotype for each gene are from the Mouse Genome Informatics database (https://www.informatics.jax.org/). AC, adenylyl cyclase; GO, gene ontology.

To enhance our focus on specific cardiac functions known to be cAMP regulated (*e.g.* heart contraction (GO:0008016) and Ca^2+^ ion transport (GO:0051924)), we analyzed the AC proximal proteins within these two GO terms (SS ≥ 0.7) (Fig. 3). Most of the listed genes have cardiac contractility, hypertrophy or developmental abnormalities upon deletion. Notably, AC5 selectively labeled the platelet-derived growth factor receptor alpha, along with a downstream negative regulator (RASA3) and potential target (MARCKSL1) of PDGFRα; all play roles in cardiac development and adult heart regeneration. AC5 also labeled proteins important in calcium handling, including junctophilin (JPH2), WFS1, and calsequestrin 2 (CASQ2) (both AC5 and AC6). CASQ2 is a regulator of the RYR2, which is required for excitation coupling in CMs and stabilized by JPH2-mediated junctional complexes between the PM and SR. Although AC9 labeled few selective cardiac proteins, its proximity to AKAP5 and SNTA1 (and weak labeling of KCNQ1, SS = 0.5) is in accord with its roles in cardiac repolarization and inheritable arrhythmias (19, 29, 30).

### Cluster analysis of trafficking-associated near-neighbor prey proteins using STRING

**Figure 4:**
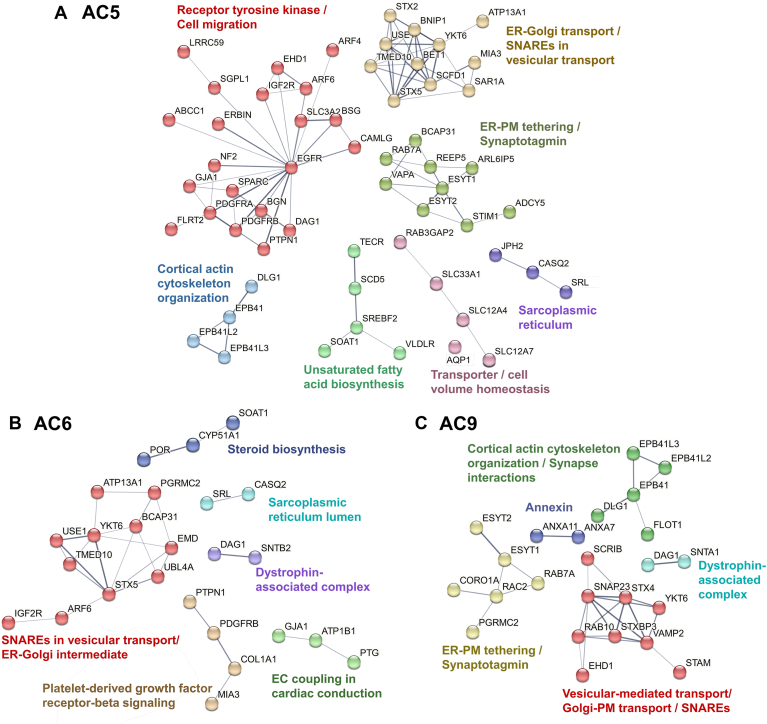
**The cluster analysis of prey proteins using STRING.***A–C*, for each AC isoform, *solid lines* indicate connection within a cluster, with line thickness representing the strength of supporting data. Specific terms assigned to each cluster are shown. AC, adenylyl cyclase.

Protein localization and protein folding were among the top biological processes for ACs based on gProfiler. AC isoforms have been detected at the PM, as well as at endomembranes (31, 32); however, little is known about the trafficking of the major cardiac ACs, in CMs. Thus, we performed STRING analysis on AC5, AC6, and AC9 prey proteins (SS ≥ 0.7) found within localization (GO:0051179) and endomembrane (GO:0012505) terms (excluding protein folding related proteins (GO:0006457), which included much of the cellular response to ER stress for AC5) (Fig. 4). Next, the Markov cluster algorithm within STRING was used to classify groups. In addition to the clusters mentioned above, AC5/6 shared many proteins associated with SNAREs and ER-Golgi vesicle transport, while AC5 and AC9 labeled proteins associated with ER-PM tethering and cortical actin cytoskeleton organization. AC9 also labeled a unique group of SNAREs and Golgi-PM transport proteins, in addition to annexin family members involved in vesicle trafficking and membrane dynamics. Thus, AC9 appears to encounter a different set of trafficking proteins than AC5/6, including RAB small GTPases and v- and t-SNAREs that regulate vesicular transport to endocytic vesicles, vesicle movement, and endosome fusion (33). This is consistent with AC9 signaling from endosomes (31, 32).

### Complementation of BioID with FLAG-pulldown mMS

**Figure 5:**
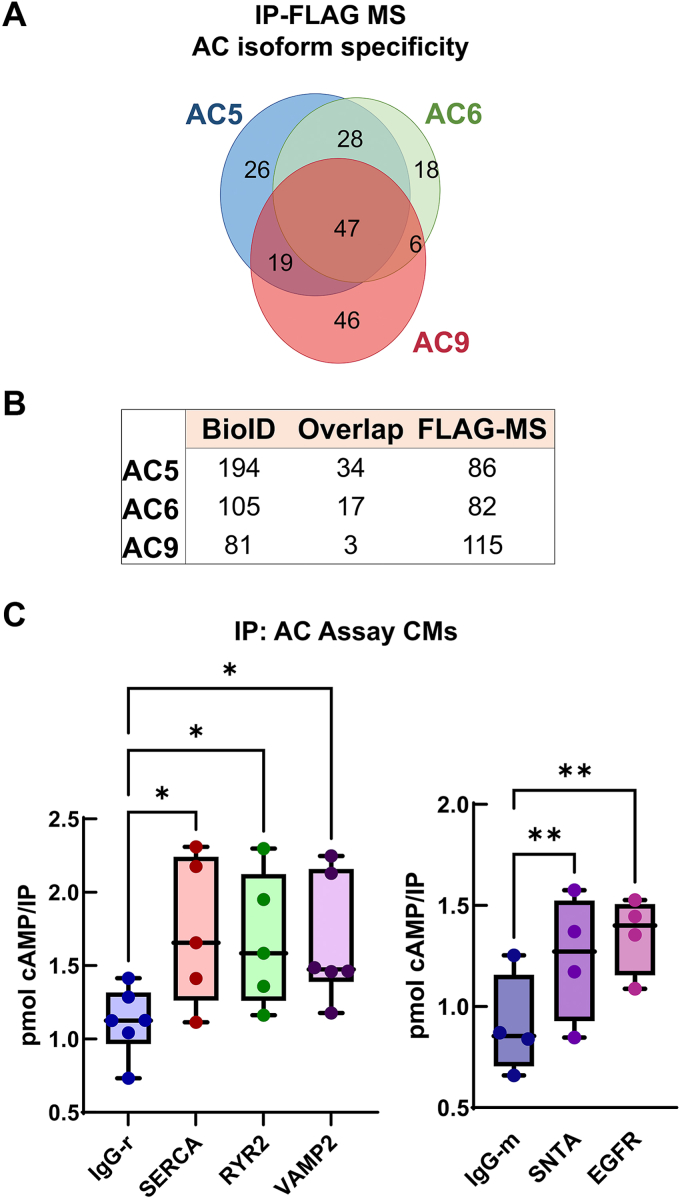
**Comparison of proximity datasets for ACs obtained with BioID and FLAG-IP approaches.***A*, Venn diagram of the FLAG-MS datasets for the three AC isoforms (SS ≥ 0.7). *B*, the total number of unique and shared proteins identified in the BioID and FLAG-MS datasets for each AC isoform (SS ≥ 0.7). *C*, endogenous AC activity pulls down with novel BioID and/or FLAG-MS identified proteins. CMs were subjected to coimmunoprecipitation with antibodies against the indicated proteins and assayed for AC activity with 300 nM Gαs or 50 μM Fsk. Box-whisker plots indicate mean and full data range, n = 4 to 6 experiments, performed in duplicate. Statistics: ∗*p* < 0.05; ∗∗*p* < 0.01. One-way ANOVA, Dunnett’s multiple comparison test compared to IgG-rabbit or IgG-mouse control group. AC, adenylyl cyclase; BioID, biotin identification; CM, cardiomyocyte; MS, mass spectrometry.

To complement the BioID analysis, we employed a second approach, FLAG-pulldown combined with MS (FLAG-MS), to identify AC protein complexes. After eliminating nonspecific proteins (identified in GFP-mT-FLAG as a negative control), the two independent biological replicates were pooled and analyzed using SAINT with a filter of SS ≥ 0.9 to identify interacting proteins (Table S2). The FLAG datasets for the different AC isoforms showed a similar degree of overlap to that of the BioID datasets (Figs. 2*D* and 5*A*), although more total proteins were shared by all three isoforms. Comparisons of the FLAG-MS and BioID datasets indicated that ∼15% of proteins were shared between the two techniques for AC5 and AC6, while only ∼4% were shared for AC9 (Fig. 5*B*). This is not entirely unexpected, as some protein interactions may be too weak or transient to withstand the FLAG pull-down procedure, be poorly solubilized by the detergents used for FLAG-MS, or outside the distance constraints or lack exposed lysines required for BioID. Most shared proteins were involved in protein folding or trafficking. To determine if BioID and FLAG-MS identified different proteins within a given cluster, STRING analysis was performed using the combined datasets for terms corresponding to protein trafficking (Fig. S4*A*). There was clear overlap within a given cluster identified by the two techniques, suggesting that there is an advantage to using both experimental paradigms.

### Validation of endogenous AC interactions by IP-AC assay in CMs

**Figure 6:**
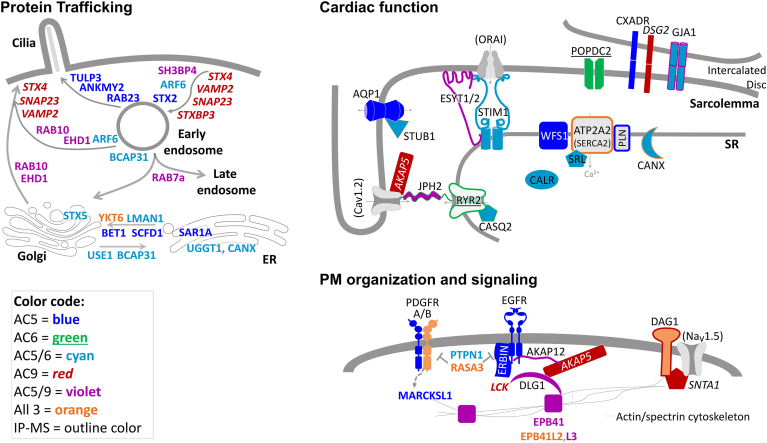
**AC proximal interactomes in neonatal cardiomyocytes.** The schematic shows the AC complex map, based on current knowledge of trafficking, cardiac function, and plasma membrane signaling complexes. The proteins identified as AC proximal complexes (SS ≥ 0.7) are color coded with respect to the individual AC isoform (see legend for details). *Shape outline colors* represent FLAG-MS identified proteins (SS ≥ 0.9); those filled *gray* (*i.e.,* RYR2 and ATP2A2) were identified by FLAG-MS alone. AC, adenylyl cyclase; MS, mass spectrometry; RYR2, ryanodine receptor 2.

We selected several specific proteins for further characterization that were identified by BioID and/or FLAG pulldowns with known cardiac functions and had available antibodies for immunoprecipitation of endogenous proteins. These included VAMP2, SERCA2 (ATP2A2), RYR2, SNTA1, and EGFR. We performed immunoprecipitations (IP) from neonatal CMs followed by measurement of associated endogenous AC activity (IP-AC assay), as this is a far more sensitive and rigorous assay for identifying functional AC complexes. Endogenous AC activity was significantly associated with VAMP2, SERCA2, RYR2, SNTA1, and EGFR compared with control IgG (Figs. 5*C* and S4*B*). EGFR coimmunoprecipitation with endogenous AC5 was confirmed by Western blot (S4B). Based on the FLAG-MS, BioID datasets, and our validated AC-associated proteins, we developed a proximity map (Fig. 6) that focused on proteins involved in the trafficking of membrane-bound organelles, cardiac contractile function, and PM organization, and signaling. We further incorporated overlap of AC datasets with the PDE interactome from CMs from Subramaniam *et al.* (Fig. S5, (7)). These authors identified cyclic AMP-dependent phosphoproteomes and interactomes associated with three cardiac PDE isoforms. Our BioID and FLAG-MS analysis of AC isoforms identified several overlapping proteins including, RYR2 (AC6 FLAG-MS; PDE2/PDE3), SERCA2 (all ACs by FLAG-MS; PDE3A), JPH2 (all ACs; PDE3A1), and CASQ2 (AC5/6; PDE3A1 and 3A2), indicating shared roles in calcium handling. Additionally, several trafficking-related proteins were identified exclusively by AC9 and PDE3A1 (Fig. S5). The details of these complexes are discussed below, but overall our BioID and FLAG-MS approaches have successfully identified proteins proximal to specific AC isoforms in CMs.

### Heterodimerization of AC5 and AC6

**Figure 7:**
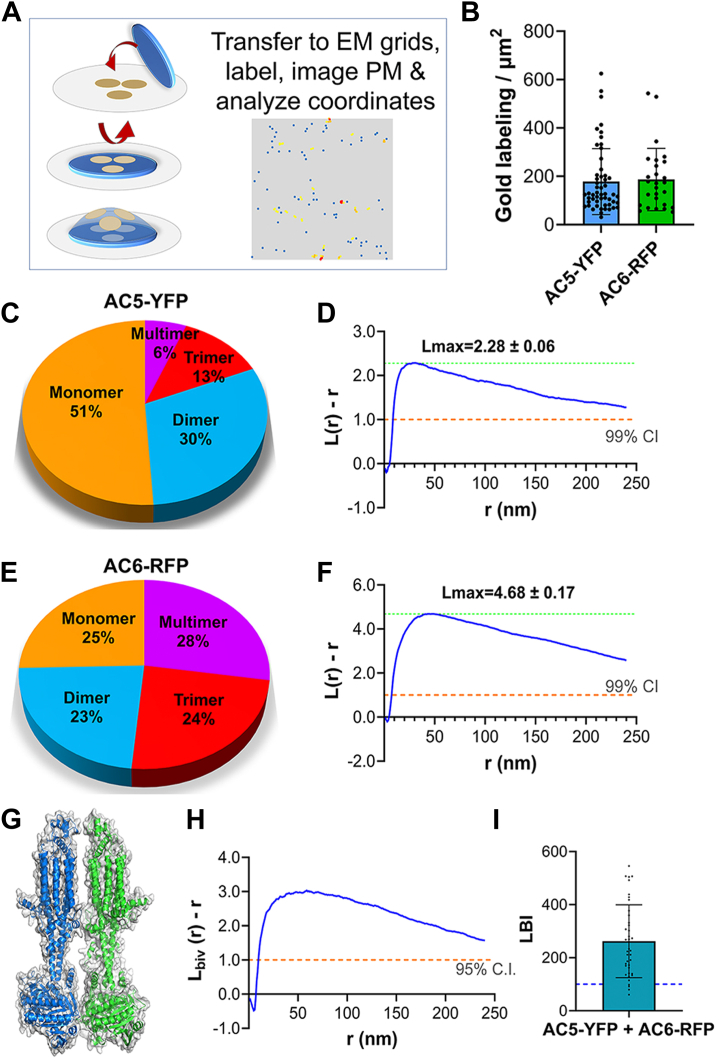
**Super-resolution electron microscopy quantitates the spatial distribution of AC5 and AC6 on intact plasma membrane sheets.***A*, schematic representation of EM-based spatial analysis. *B*, PM localization of AC5 or AC6. Intact PM sheets from the BHK cells were attached to EM grids and immunolabeled with anti-YFP (AC5) or anti-RFP (AC6) antibody conjugated to gold particles (4.5 nm). *C* and *E*, relative proportions of monomers, dimers, and higher order oligomers (nanoclusters) on the PM for AC5 and AC6, as estimated by local L(r) – r analysis. *D* and *F*, Ripley’s K-function calculates the spatial distribution of the gold particles shown in (*B*). Extent of nanoclustering, L(r) – r, is plotted as a function of radius r in nanometer. L(r) – r values above the 99% confidence interval (99% CI) indicate statistically significant nanoclustering. The peak L(r) – r value, Lmax, is used as a summary statistic to quantify the extent of clustering. *G*, heterodimer model of AC5 (*blue*) and AC6 (*green*) was generated using the AC5 dimer structure (PDB: 8SL4) and superimposing chain B with AC6 (modeled using AlphaFold; aa 1–110 not shown). *H*, Ripley’s bivariate colocalization K-function analysis. Extent of coclustering, L_biv_(r) – r, is plotted as a function of radius r in nanometer, where values above the 95% confidence interval (95% CI) indicate statistically significant coclustering. *I*, statistical summary of coclustering. Integration of the L_biv_(r) – r curve (between r = 10–110 nm) yields a statistical summary, termed L-bivariate integrated (LBI). Each point represents analysis of an individual 1 μm^2^ EM image (from 3 to 4 independent experiments). AC, adenylyl cyclase; EM, electron microscopy; PM, plasma membrane.

The overlap in BioID and FLAG-MS–interacting proteins between AC5 and AC6 suggested that these two isoforms have interchangeable roles and/or may even form heterodimers (Figs. 2*D* and 5*A*). Importantly, AC5 and AC6 datasets contained several unique peptides for the other isoform by BioID and by FLAG-MS (Fig. S6*A*). To confirm the presence of homodimers and/or heterodimers of AC5/6, we performed EM spatial mapping. BHK cells were transfected with AC5-YFP or AC6-RFP. Intact apical PM sheets were attached to EM grids, immunolabeled with anti-YFP or anti-RFP antibody conjugated to gold nanoparticles (4.5 nm), and images were acquired *via* transmission EM at 100,000× magnification (Fig. 7*A*). AC5 and AC6 showed similar incorporation into the PM with mean gold labeling of 169 and 186 particles/μm^2^, respectively (Fig. 7*B*). The univariant nanoclustering of immunogold particles was measured by Ripley’s univariate K-function analysis (Figs. 7, *C*–*F*, and S6*B*). The extent of nanoclustering, L(r) – r, was plotted as a function of distance, r (Figs. 7, *D* and *F*, and S6*B*). The peak value of the L(r) – r curves, Lmax, serves as a statistical summary of nanoclustering, with values greater than the 99% confidence interval (99% CI) of 1.0 considered statistically meaningful clustering (Fig. 7, *D* and *F*). Both AC5 and AC6 exhibit significant coclustering, with AC5 existing as both monomers and dimers, while AC6 was found more often as dimers, trimers, and higher ordered multimers (Fig. 7, *C* and *E*). To determine the degree of AC5:AC6 heterodimerization (modeled in Fig. 7*G*), AC5-YFP and AC6-RFP were coexpressed and labeled with 6 nm and 2 nm gold particles coupled to anti-GFP and anti-RFP, respectively. The coclustering between the two populations of gold particles (6 nm and 2 nm) were quantitated with the Ripley’s bivariate K-functions. Extent of coclustering, L_biv_(r) – r, was plotted against distance r with the defined integral of the function, termed L-function bivariate integrated (LBI), serving as a summary parameter (Fig. 7, *H* and *I*). The LBI values above the 95% CI of 100 indicate statistically meaningful coclustering, or heterodimerization (Fig. 7*I*). The larger the LBI value, the more extensive the colocalization of the 2 nm and 6 nm fold patterns, with AC5/6 displaying a significant LBI of 262 ± 22 (Fig. 7*I*), consistent with the cryo-EM structure of an AC5 homodimer (34) and the overlap in BioID datasets for AC5/6.

## Discussion

AC nanodomains have been largely undefined in CMs or any cell type, with the exception of interactions with AKAPs (14). Using an unbiased BioID proteomics approach, we identified the near-neighbor proximity proteins for three cardiac AC isoforms. AC5 and AC6 are the major AC isoforms in heart with many shared properties, consistent with our observation that ∼87% of AC6 proximal proteins are shared by AC5 and that AC5/AC6 form heterodimers. However, AC5 and AC6 display distinct phenotypes when deleted and their expression is differentially regulated with respect to development, age, and stress responses (35, 36). AC9 is expressed at much lower levels but plays important roles in baseline cardiac stress responses and regulation of heart rate and cardiac repolarization (18, 19). AC9 is quite distinct from other ACs, a feature mirrored in its proximal proteins, overlapping only 48% and 21% of proximal proteins with AC5 and AC6, respectively. These proximal datasets highlight important roles in trafficking, intracellular signaling, and cardiac function as discussed below.

### AC interaction networks in cardiac contractility, calcium handling, and stress responses

GO enrichment analysis of terms for Ca^2+^ homeostasis and cardiac contractility led to several emerging patterns. Both AC5/6 regulate aspects of calcium handling in heart, consistent with their proximity to numerous ER/SR proteins (Fig. 2*C*). For example, AC6 was found proximal to the RYR2 (in FLAG-MS datasets), while AC5 was associated with regulatory proteins involved in excitation–contraction coupling, including JPH2, CASQ2, and weakly AKAP5 (SS = 0.5). This is consistent with AC5 decreased isoproterenol-stimulated left ventricular ejection fraction observed upon *Adcy5* deletion in mice (37) and its role in β2-AR enhancement of L-type Ca^2+^ currents (16).

Deletion of *Adcy6* decreases the Ca^2+^ affinity of SERCA2a by 3.5-fold and reduces caffeine-stimulated Ca^2+^ transients by 50% (38), suggesting that AC6 may be the primary regulator of calcium reuptake. However, both AC5 and AC6 pulled down SERCA2 (ATP2A2) machinery for calcium reuptake into the SR. Conversely, antibodies against SERCA2 pulled down endogenous AC activity, although we could not distinguish which AC isoform. Several regulators of SERCA2 were identified by BioID, including sarcalumenin (both AC5/6) and wolframin (WFS1; AC5). Although PLN, which mediates PKA regulation of SERCA2, did not reach significance by BioID due to high background labeling, it was selectively pulled down by FLAG-tagged AC5. Thus, both AC5/6 appear to be proximal to SERCA2 complexes in neonatal CMs. Finally, both AC5/6 appear proximal to STIM1, the calcium sensing, and regulator of store-operated ORAI1 channels (not labeled).

AC5 has long been associated with cardiac stress responses. Deletion of AC5 is protective against chronic β-AR stimulation, chronic pressure overload, and age-related cardiac myopathy (39, 40, 41). AC5 selectively labeled proteins involved in physiological hypertrophy (42), including the receptors EGFR and platelet-derived growth factor receptor alpha, and the cardioprotective ERBB2-interacting protein (ERBIN). Moreover, immunoprecipitation of EGFR from CMs pulled down endogenous AC activity, which was confirmed as AC5 by Western blotting. AC/cAMP/PKA and EGFR have a long history of cross-talk, which may be facilitated by localized signaling. PKA promotes EGFR endocytosis and transactivation, while EGFR signaling can influence PKA activity (43, 44, 45). The downstream negative regulators (RASA3 and PTPN1) and a potential downstream target (MARCKSL1) were also labeled, which have cardiac development and/or regenerative roles. AC5 was also proximal to the PKA scaffolding protein AKAP12, along with the discs large MAGUK-scaffold protein 1 (DLG1) which may help to assemble stress-responsive nanodomains for cardiac hypertrophy.

The modest changes we observed in AC interactomes following chronic NE stimulation were surprising given the well-established role of NE in driving cardiac hypertrophy and relocalization of β-ARs in heart failure models (1, 17). Despite the few changes, NE-induced proximal proteins for AC5 play key roles in cardiac function. For example, SPARC is increased upon hypertrophy and regulates extracellular matrix and CM cell shortening (46). AC6 proximal protein prostacyclin synthase is also upregulated upon hypertrophy and heart failure, while ATP1B1 encodes the beta subunit of the sodium potassium ATPase which is essential for the myocardial resting membrane potential and is often associated with cardiovascular disease (47, 48). Fine-tuning of localized regulation may allow CMs to recalibrate signaling dynamics in response to chronic stress without globally restructuring the AC-associated proteome.

The few contractile-related proteins labeled by AC9 were associated with cardiac arrhythmias, including SNTA1 and desmoglein (DSG2). AC9 weakly labeled KCNQ1 (SS = 0.5 for Veh and NE conditions), which encodes the delayed rectifier potassium I_Ks_ channel that is associated with long QT syndrome and requires AC9 for β-AR stimulation (29). Deletion of *Adcy9* in mice leads to decreased heart rate at rest, enhanced heart rate variability upon recovery from stress, and decreased isoproterenol-stimulated phosphorylation of KCNQ1 and I_KS_ currents (19, 29, 49). Mutations in the sodium channel Nav1.5 or its regulators can also lead to cardiac arrhythmias. AC9 proximal proteins SNTA1 and dystroglycan (DAG1) regulate Nav1.5 currents and are linked to arrhythmia-prone long QT syndrome, Brugada’s syndrome, and muscular dystrophy, respectively (50). The enhanced trafficking of Nav1.5 by cAMP/PKA begs the question of whether AC9 may be partially responsible for sympathetic regulation of sodium currents in heart (51).

### Evidence for heterodimerization of AC5 and AC6

There was significant overlap between AC5/6 proximal and FLAG-MS datasets. This may not be surprising given their regulatory and sequence similarity (15). In addition, AC5 and AC6 labeled one another (appearing in the top 10 labeled prey for each) and were present in the FLAG-MS datasets for the corresponding isoform. AC5 weakly appeared in the FLAG-MS set for AC9, but the reverse was not observed. AC isoforms have previously been reported to form homodimers based on numerous approaches (25, 52, 53, 54, 55). Importantly, an AC5 homodimer was observed by cryo-EM (34); however there has only been weak evidence for AC heterodimers (25, 56). EM spatial mapping shows clear evidence for AC5 and AC6 homodimerization, while also indicating the existence of AC5:AC6 heterodimers. Thus, our data present both visual and functional evidence for heterodimerization of AC5/6, which likely drives the large overlap between AC5/6 proximal datasets.

### Localization of AC near-neighbor proteins: roles in intracellular signaling and trafficking

Compartmentalization analysis of AC proximal proteins found enrichment at multiple subcellular sites, including PM, Golgi, ER/SR, and along the endocytic trafficking pathway. Many of the ER resident proteins are likely involved in the folding and glycosylation of AC proteins and their subsequent trafficking to the PM. However, accumulating evidence suggests that internalized GPCR and G protein subunits are required to initiate a second wave of cAMP signaling from endomembrane sites (57, 58, 59). Moreover, the site of signal origination can have profoundly different functional outcomes. For example, Golgi-localized GPCRs enhance cAMP production and CM relaxation (60, 61) or even hypertrophy (62, 63), while those on the PM drive CM contraction. However, few studies have identified functional GPCR-activated ACs at intracellular sites. AC activity has been detected in isolated Golgi from liver (64), but only AC9 is known to transit to endosomes and mediate endosomal GPCR-initiated cAMP production (31). This is consistent, with AC9 labeling of numerous proteins that regulate the fusion and fission cycles of endocytosis from the PM to the Golgi, through early and late endosomes (33, 65). AC9 labeling of VAMP2 (also known as synaptobrevin 2, a core SNARE protein located on synaptic vesicles) may be related to its association with POPDC2, which is required for transferrin and integrin receptor recycling and TREK-1 trafficking (19, 66). We found VAMP2 associated with endogenous AC activity, consistent with its known roles in cAMP-regulated recycling and translocation of many ion channels and transporters (67, 68, 69).

AC5 labeled a subset of the same endocytic regulators as AC9, suggesting that it too may facilitate cAMP production from endomembranes. Notably, all 3 ACs show perinuclear localization in neonatal CMs that partially overlaps with the *trans*-Golgi network. Finally, AC5/6 suppress Hedgehog signaling (70), localizing to cilia *via* interactions with ANKMY2 (71). AC5 labeled ANKMY2, along with seven members of the smoothened signaling pathway (GO:0007224), including ciliary GPCR-trafficking protein TULP3 (72).

### Limitations and distance constraints of the study

Both BioID and FLAG-MS failed to identify some known cardiac AKAP interactors (*e.g.* Yotiao and mAKAP (73, 74)), GPCRs, and ion channels and transmembrane proteins that form complexes with ACs (*e.g.* Cav1.2 and BVES). These missing protein interactions could be due to poor biotinylation, solubilization, and/or MS detection of these membrane-associated or transmembrane proteins. Another issue could be due to the C-terminal location of the mT tag which places the BirA enzyme ∼76 Å off the membrane (26) and possibly outside the distance constraints of labeling. Complementary methods and/or alternate fusion protein constructs may be required to identify these types of interactions, particularly in the case of transmembrane proteins. Unexpectedly, PDEs were not labeled or pulled-down by BioID or FLAG-MS for any AC isoform. Similarly, Subramaniam *et al.,* (7) did not identify ACs in pulldowns of PDE2A2, PDE3A1, or PDE3A2 from CMs. Due to technical feasibility, both our study (BioID and FLAG-MS) and that of Subramaniam (mCherry-MS) utilized neonatal CMs, which have a less developed SR and T-tubule network and thus differ in their reliance on excitation–contraction coupling. Nevertheless, a subset of proteins involved in cardiac function were identified by both PDEs and ACs, including those associated with excitation–contraction coupling in the SR (RYR2, JPH2, and CASQ2), SERCA2 calcium reuptake (ATP2A2, sarcalumenin, PLN, CALR), and gap-junction and adhesion proteins at the intercalated disc. ACs and PDEs are important regulators of calcium handling processes so one would expect them to reside in overlapping complexes related to these activities.

In closing, recent studies have challenged the classic paradigm that AC-dependent regulation relies on activation of ACs on the PM and subsequent diffusion of cAMP and active PKA subunits to specific organelles. Our study provides a comprehensive view of interactions for AC isoforms using two independent techniques. Enrichment analysis identified potential protein interactions involved in cardiac contractility and trafficking pathways. Furthermore, we have validated five cardiac proximal proteins (SNTA1, VAMP2, RYR2, ATP2A2, and EGFR) and show their association with endogenous AC activity. Finally, EM spatial mapping provides visual evidence of AC5/6 heterodimerization, which likely drives much of the overlap between proximal proteins for these two AC isoforms. Thus, our proximity dataset serves as an important resource for exploring function of ACs in cardiac contraction and relaxation and identifies trafficking-related proteins for specific AC isoforms.

## Experimental procedures

### Antibodies and reagents

Antibodies used for immunoprecipitation, Western blotting, immunocytochemistry, and proximity ligation assay (PLA) are listed in Table 1. Mouse anti-AC5 antibody was generated and characterized as described (75). Norepinephrine (Sigma) was stored and diluted in AT buffer (100 mM ascorbate and 10 mM thiourea, pH 7.4). Forskolin (MP Biomedicals, #190669) was stored in dimethyl sulfoxide.

**Table 1:** Antibodies and dilutions

Antibody	Company/Catalog number	Dilution
Mouse anti-DYKDDDDK (FLAG) M2	Sigma-Aldrich/F3165	1:1000
Rabbit IgG	Cell Signaling/#2729	1:1000
Mouse IgG	Santa Cruz/sc-2025	1:1000
Mouse anti-Alpha-actinin (sarcomeric)	Sigma-Aldrich/A7811	1:500
Rabbit anti-MYBPC3	Proteintech/19977-1-AP	1:300
Rabbit anti-phopho-PKA substrate (RRXS∗/T∗) (100G7E)	Cell Signaling/#9624	1:500
Streptavidin, horseradish peroxidase–conjugated	Invitrogen/S911	1:2000
Alexa 488 anti-mouse	Invitrogen/A21202	1:500
Alexa 488 anti-rabbit	Invitrogen/A21206	1:500
Alexa 647 anti-mouse	Invitrogen/A32787	1:500
Alexa 568 anti-rabbit	Invitrogen/A10042	1:500
Mouse anti-beta-actin	Santa Cruz/sc-47778	1:3000
Mouse ant-PKA RIIβ	BD Biosciences/610,625	1:1000
Rabbit anti-G β (T-20)	Santa Cruz/sc-378	1:1000
Rabbit anti-ATP2A2	Cell Signaling/#9580S	1:300
Rabbit anti-RYR2	Alomone labs/ARR-002	1:300
Rabbit anti-VAMP2	Abcam/ab215721	1:300
Mouse anti-SNTA1	Abcam/ab11425	1:300
Mouse anti-EGFR	Santa Cruz/sc-373746	1:300

EGFR, epidermal growth factor receptor; RYR2, ryanodine receptor 2; SNTA1, syntrophin alpha 1; VAMP2, vesicle-associated membrane protein 2.

### Virus and plasmids

The constructs for human AC-mT-FLAG and GFP-mT-FLAG were generated by PCR and subcloned into the vector pCDNA3 (accession numbers *Adcy5*, NM_183357; *Adcy6*, NM_015270.5; *Adcy9*, NM_001116.4). All constructs contain the gene of interest, followed by a short linker (13 amino acids), the BirA variant mT (22), and a C-terminal FLAG tag. Each construct was verified by DNA sequencing using forward, reverse, and internal primers. The 1.6 to 4.8 kilobase inserts were subsequently subcloned into the vector pDUAL-CCM(+) and adenoviruses (Ad5 serotype with E1/E3 deletion) were generated by Vector Biolabs (Malvern). Adenoviruses were amplified in HEK293 cells, filtered using 0.45 um filters to remove cellular debris, titered using the QuickTiter Immunoassay Kit (VPK-109, Cell BioLabs), aliquoted, frozen, and stored at −80 °C.

### Isolation, culture, and infection of CMs

All procedures were approved by the McGovern Medical School Institutional Animal Care and Use Committees. Neonatal CMs were isolated enzymatically from 2- to 3-day-old Sprague-Dawley rat heart and enriched *via* percoll gradient centrifugation. Briefly, hearts were extracted by enzymatic reaction of collagenase (73 U/ml) and pancreatin (0.6 mg/ml) with ADS buffer (116 mM NaCl, 20 mM Hepes, 0.8 mM NaH_2_PO_4_, 5.6 mM glucose, 5.4 mM KCl, 0.8 mM MgSO_4_, pH 7.4, and 0.22 μm filtered). Cells were layered onto a percoll gradient (densities: 1.050, 1.060, and 1.082 g/ml) and subjected to centrifugation (1000*g* for 30 min). Isolated CMs were seeded onto cell culture plates and cultured in medium containing Dulbecco's modified Eagle's medium-F10 with 10% horse serum, 5% fetal bovine serum, 1% penicillin/streptomycin at 37 °C in a humidified atmosphere containing 5% CO_2_. Neonatal CM purity was evaluated by immunocytochemistry with alpha-actinin (staining for Z disk in sarcomeres) and phalloidin (staining for F-actin in actin filaments) and shown to be over 90%. For confocal imaging, glass coverslips and/or chambers were precoated with 15 μg/ml of laminin and 100 μg/ml of poly-D-lysine (MilliporeSigma, MD). Medium was changed 24 and 48 h post isolation. CMs were infected with adenoviruses 48 h post isolation) with the following multiplicity of infections (GFP-mT = 8; AC5- and AC6-mT = 80; and AC9-mT = 10) to obtain similar levels of expression (Fig. S1*A* for timeline of treatments).

### *In vitro* hypertrophy and cell size measurements

CMs (48 h postinfection) were incubated with 10 μM norepinephrine or vehicle daily for 2 days to induce hypertrophy. The mRNA expression of hypertrophic genes BNP and ANP were measured by RT-PCR using the following primers: *BNP*, 5′-ACA ATC CAC GAT GCA GAA GCT and 5′-GGG CCT TGG TCC TTT GAG A; and *ANP* 5′-ATC TGA TGG ATT TCA AGA ACC and 5′-CTC TGA GAC GGG TTG ACT TC. The fold change in transcript levels (2^ΔΔCt^) between NE and vehicle control samples was calculated from the Cts of each primer set as follows, where ΔΔCt = ΔΔCt (ANP_NE_ – GAPDH_NE_) – ΔCt(ANP_Veh_–GAPDH_Veh_). To measure changes in cell size, cells were cultured on MatTEK plates (MatTek) coated with 15 μg/ml of laminin and 100 μg/ml of poly-D-lysine. Cells were treated with hypertrophic agents for 48 h and stained with the CM marker, α-actinin, and the nuclear marker Hoechst dye (Sigma, 94403). Changes in cell size were quantitated by ImageJ (Ver.1.52p; imagej.net/software/fiji/).

### MS sample preparation for proximity proteomics

Adenoviral-infected CMs were treated with 50 μM biotin (Sigma, B4501) for 3 h, rinsed, scraped with PBS, and frozen in liquid N_2_. Cell pellets were prepared for MS analysis by the NBCC Proteomics Core at the Lunenfeld-Tanenbaum Research Institute as described (76). Frozen cell pellets were lysed in 1:10 (cell pellet weight:lysis buffer) using RIPA lysis buffer (50 mM Tris–HCl (pH 7.5), 150 mM NaCl, 0.1% (w/v) SDS, 1% NP-40, 1 mM MgCl_2_, 1 mM EDTA, 0.5% (w/v) sodium deoxycholate, and 1× Sigma protease inhibitors). The lysate was sonicated (3 × 5 s, 2 s off) at 30% amplitude using a “1/8” microtip. TurboNuclease (250 units) and 10 μg RNase was added to each sample and incubated, with rotation, at 4 °C for 30 min. Additional SDS was added to bring the final concentration to 0.25% SDS, and the samples were centrifuged at 14,000 rpm for 20 min at 4 °C. The lysate was applied to 30 μl of prewashed streptavidin beads (Cytiva 17-5113-01) and incubated at 4 °C, rotating, for 3 h. After incubation, supernatant was removed and beads were washed one time with 2% SDS, 50 mM Tris–HCl pH 7.5, two times with RIPA lysis buffer, and three times with 50 mM ammonium bicarbonate.

Proteins on beads were digested with 1 μg of trypsin in 50 μl of 50 mM ammonium bicarbonate, overnight at 37 °C. Peptides were moved to a new tube, beads were washed with 50 μl water, and this wash was combined into the same tube. Additional trypsin (0.5 μg) was added and incubated at 37 °C for 4 h. Formic acid was added to a final concentration of 5%, peptides were desiccated and stored at −40 °C until MS analysis.

### FLAG affinity purification for MS analysis

Frozen cell pellets were resuspended in 400 μl ice-cold lysis buffer (50 mM Hepes-NaOH pH 8.0, 100 mM KCl, 2 mM EDTA, 0.1% NP-40, 10% glycerol, 1 mM PMSF, 1 mM DTT, and 1X protease inhibitor cocktail). Cells were sonicated three times for 5 s on ice, followed by addition of 250 U of TurboNuclease and 10 μg of RNase for 15 to 20 min at 4 °C. Samples were subjected to centrifugation at 20817*g* for 20 min, and the supernatant was removed and used for subsequent pull downs.

Anti-FLAG M2 magnetic beads (25 μl of a 50% slurry per sample) were washed in lysis buffer then incubated with clarified lysate for 2 to 3 h at 4 °C. Beads were washed once with lysis buffer, twice with (20 mM Tris–HCl pH 8.0, 2 mM CaCl_2_; 1 ml each time), and resuspended in 7.5 μl of trypsin digestion buffer (100 ng/μl trypsin in 20 mM Tris–HCl (pH 8.0) and processed as described above prior to analysis by nano-HPLC coupled to MS.

### MS analysis

For data-dependent acquisition (DDA) LC-MS/MS, affinity purified and digested peptides were analyzed using a nano-HPLC coupled to MS. All of the samples were used. Nano-spray emitters were generated from fused silica capillary tubing, with 100 μm internal diameter, 365 μm outer diameter and 5 to 8 μm tip opening, using a laser puller (Sutter Instrument Co., model P-2000, with parameters set as heat: 280, FIL = 0, VEL = 18, DEL = 2000). Nano-spray emitters were packed with C18 reversed-phase material (Reprosil-Pur 120 C18-AQ, 3 μm) resuspended in methanol using a pressure injection cell. Sample in 5% formic acid was directly loaded at 800 nl/min for 20 min onto a 100 μm × 15 cm nano-spray emitter. Peptides were eluted from the column with an acetonitrile gradient generated by an Eksigent ekspert nanoLC 425, and analyzed on a TripleTOF 6600 instrument (AB SCIEX). The gradient was delivered at 400 nl/min from 2% acetonitrile with 0.1% formic acid to 35% acetonitrile with 0.1% formic acid using a linear gradient of 90 min. This was followed by a 15 min wash with 80% acetonitrile with 0.1% formic acid, and equilibration for another 15 min to 2% acetonitrile with 0.1% formic acid. The total DDA protocol is 135 min. The first DDA scan had an accumulation time of 250 ms within a mass range of 400 to 1800 Da. This was followed by 10 MS/MS scans of the top 10 peptides identified in the first DDA scan, with accumulation time of 100 ms for each MS/MS scan. Each candidate ion was required to have a charge state from 2 to 5 and a minimum threshold of 300 counts per second, isolated using a window of 50 mDa. Previously analyzed candidate ions were dynamically excluded for 7 s.

### SAINT and network analysis

MS data generated were stored, searched, and analyzed using ProHits laboratory information management system platform. Within ProHits, WIFF files were converted to an MGF format using the WIFF2MGF converter and to an mzML format using ProteoWizard (V3.0.10702; proteowizard.sourceforge.io) and the AB SCIEX MS Data Converter (V1.3 beta; sciex.com). The data were then searched using Mascot (V2.3.02; matrixscience.com) and Comet (V2016.01 rev.2; data.noaa.gov/cedit). The spectra were searched with the rat sequences in the RefSeq database (version 97) acquired from NCBI, supplemented with “common contaminants” from the Max Planck Institute (http://maxquant.org/contaminants.zip) and the Global Proteome Machine (GPM; ftp://ftp.thegpm.org/fasta/cRAP/crap.fasta), forward and reverse sequences (labeled “gi|9999” or “DECOY”), sequence tags (GFP, BirA, GST26, and mCherry), streptavidin, and LYSC_PSEAE for a total of 134,277 entries. Database parameters were set to search for tryptic cleavages, allowing up to two missed cleavages sites per peptide with a mass tolerance of 35 ppm for precursors with charges of 2+ to 4+ and a tolerance of 0.15 amu for fragment ions. Variable modifications were selected for deamidated asparagine and glutamine and oxidized methionine. Results from each search engine were analyzed through the Trans-Proteomic Pipeline (v.4.7 POLAR VORTEX rev 1) *via* the iProphet pipeline (77). Proteins were filtered based on iProphet probability ≥ 0.95 and unique peptides ≥ 2. The SAINT analysis tool was used to identify high-confidence protein interactors *versus* control samples (78). SAINTexpress (version 3.6.1 for BioID and version 3.6.3 for FLAG-AP) was used to calculate the probability that identified proteins were enriched above background contaminants. SAINTexpress uses a semisupervised spectral counting model that compares the detection of putative proximal interactors in a BioID profile of a given bait against a series of negative control runs. Bait proteins (*e.g.,* ACs and GFP) were profiled using independent biological triplicates and analyzed alongside 14 independent negative controls (no infection and GFP-mT). SAINTexpress (version 3.6.3) was used to calculate the probability that identified proteins were enriched above background contaminants. SAINTexpress uses a semisupervised spectral counting model that compares the detection of putative proximal interactors in a BioID profile of a given bait against a series of negative control runs (78). For analysis with SAINT, samples were processed through the iProphet pipeline and filtered by probability of >0.95. Minimum of two unique peptides were required for inclusion. Bait proteins were profiled using independent biological triplicates and analyzed alongside 14 independent negative controls (no infection and GFP-mT). Negative control runs consisted of streptavidin purification from cells expressing GFP-mT only (11 replicates; this represents promiscuous biotinylation by mT) or untreated cells (3 replicates; this group represents endogenous biotinylation). Enrichment analysis was constructed based on g:GOSt in g:Profiler and GO (www.geneontology.org) databases (79, 80). Molecular Function enrichment analysis was performed using PANTHER Overrepresentation Test (Released 20230705) and correlated by false discovery rate (81). Subcellular localization analysis was performed with SubCellBarCode (82) and the UniProt Knowledgebase (UniProtKB; (83)). Nonannotated proteins or those that occupy multiple groups are classified into the unclassified group. Markov cluster algorithm clustering in STRING (ver 11.5) was used for module analysis of weighted network which is sum of the number of unique protein-protein interactions connecting the respective functions.

### Immunoprecipitation and immunoblotting

FLAG-tagged ACs were infected in neonatal CMs. After 48 h, cells were rinsed with PBS and suspended in lysis buffer (20 mM Hepes, pH 8.0, 1 mM EDTA, 1 mM MgCl_2_, 1 mM DTT, 150 mM NaCl, 0.5% C_12_E_10_, and protease inhibitors). Clarified supernatants were incubated with indicated primary antibodies at 4 °C for 2 h, followed by incubation with protein A/G agarose (Santa Cruz) for 1 h. Resin was washed three times with wash buffer (20 mM Hepes, pH 7.4, 1 mM EDTA, 1 mM MgCl_2_, 110 mM NaCl, 0.05% C_12_E_10_, and protease inhibitors). Proteins were eluted with SDS-PAGE sample buffer, heated (60 °C, 10 min), and evaluated by Western blotting using NuPAGE 4 to 12% polyacrylamide gels and polyvinylidene difluoride membrane (Thermo Fisher Scientific). Membranes were blocked in 5% bovine serum albumin (BSA) in TBS with 0.1% Tween-20, incubated with the indicated antibodies (Table 1), and signal developed using SuperSignal chemiluminescent reagent (Thermo Fisher Scientific) and Odyssey image systems (LI-COR Biosciences). Uncropped images of all Western blots are shown in Fig. S7.

### cAMP accumulation assay

Neonatal CMs were treated with 100 μM IBMX for 10 min at 37 °C prior to the addition of vehicle, isoproterenol (10 μM), or forskolin (10 μM). After 10 min, reactions were stopped by 0.1 N HCl and cAMP levels were measured by enzyme immunoassay.

### AC activity assays

PM preparations from neonatal CMs were prepared and AC activity was measured as described (84). All assays were performed for 10 min at 30 °C in a final volume of 50 μl. The final concentration of MgCl_2_ and Mg-ATP in the assay mixture was 5 mM and 250 μM, respectively. Membrane preparations were stimulated with 50 nM of GTPγS-Gαs or 10 μM of forskolin; cAMP was detected by direct cAMP enzyme immunoassay according to manufacturer’s instruction (Enzo). For IP-AC assays, IP were performed as above except that after the last wash, samples were raised in suspension buffer (20 mM Hepes, pH 7.4, 1 mM EDTA, 1 mM MgCl_2_, 0.04% C_12_E_10_, and protease inhibitors) and immediately used in AC activity assays.

### PLA assay

*In situ* PLA was performed using Duolink Proximity Ligation assay kit (Sigma-Aldrich) following the manufacturer's protocol and as described (19). Neonatal CM cells (1.8 × 10^4^ CMs per well) were cultured on clear bottom 96-well plates (Corning), infected on day 3 with adenoviruses for 48 h, and then fixed with 4% paraformaldehyde. After washing the plate 3 times with PBS, the cells were blocked (1% BSA + 0.075% Triton X100) for 1 h at room temperature and then incubated with primary antibodies overnight. Anti-mouse PLUS and anti-rabbit MINUS PLA probes were incubated for 1 h at 37 °C. Subsequent steps of ligation and amplification were according to the manufacturer's protocol. After the last wash, cells were stained with DAPI (1 μg/ml 4',6-diamidino-2-phenylindole; Bio-techne) and imaged using a high-content imaging microscope (Cellomics CX5). Data analysis of mean fluorescence intensity per cell was performed using FACS analysis software (FlowJo; www.flowjo.com). Each condition was repeated at least 3 separate experiments. To prevent false positives, cells with saturating YFP fluorescence were not considered in the analysis. Primary antibodies included: mouse anti-M2 FLAG (Sigma-Aldrich, 1:1000) and rabbit anti-Gβ (SantaCruz, 1:1000). Signal was normalized to control GFP signal to account for background variability between experiments.

### EM spatial analysis

BHK cells were seeded on glass coverslips and transfected with YFP- or RFP-tagged proteins. Approximately 18 h posttransfection, intact PM sheets from BHK cells were mounted onto copper EM grids, fixed with 4% paraformaldehyde and 0.1% glutaraldehyde, immunolabeled with 4.5-nm gold nanoparticles conjugated to anti-YFP antibodies, and subsequently negative-stained with uranyl acetate (85, 86, 87). Transmission electron microscopy was employed to capture images of the gold nanoparticles on the PM at a magnification of 100,000×. The coordinates of each gold particle were assigned using ImageJ software. The nanoclustering of gold particles within a 1 μm^2^ PM area was determined through Ripley’s K-function (85, 86). The univariate K-function analysis is utilized to quantify the lateral spatial distribution of a single population of immunolabeled gold nanoparticles on intact PM sheets (*e.g.* homodimerization analysis). This analysis evaluates the null hypothesis that the distribution of points within the selected area is random.

$$K\left(r\right)={An}^{-2}\sum_{i\neq j}\;w_{ij}1\left(\|{\text{x}}_{\text{i}}-{\text{x}}_{\text{j}}\|\leq r\right)$$

$$L\left(r\right)–r=\sqrt{\frac{K\left(r\right)}{\pi }}-r$$

K(r) represents the univariate K-function for the number of gold nanoparticles (n) within an intact PM area of A. The parameter r indicates the distance ranging from 1 to 240 nm, with increments of 1 nm. The parameter || · || is the Euclidean distance, where || · || is 1 under the condition of ||*x*_*i*_ *− x*_*j*_|| ≤ r, while || · || is 0 when ||*x*_*i*_ *− x*_*j*_|| > r. An unbiased edge correction is included in this analysis. K(r) is converted into L(r) – r and normalized against the 99% CI estimated by Monte Carlo simulations. An L(r) − r value of 0 for all corresponding r values suggests a completely random distribution of nanoparticles. An L(r) − r value exceeding the 99% CI (*i.e.,* 1.0) indicates statistically meaningful clustering at the specified length scale, with the maximal value (Lmax) providing a summary statistic that correlates with the extent of clustering. For each experimental condition, at least 20 PM sheets (1 μm^2^ area) were imaged, analyzed, and combined.

The K-function bivariate coclustering analysis computes the coclustering between two differently sized gold nanoparticles that label distinct proteins on intact PM sheets. Following a procedure similar to the univariate nanoclustering method described above, intact apical PM sheets from BHK cells coexpressing YFP-AC5 and an RFP-tagged AC6 construct were attached to EM grids, fixed with 4% paraformaldehyde and 0.1% glutaraldehyde. The PM-sheets were incubated with 6 nm gold nanoparticles conjugated to anti-YFP antibodies, blocked with 0.2% BSA and 0.2% fish skin gelatin, and then incubated with 2 nm gold nanoparticles conjugated to anti-RFP antibodies. Coordinates of the gold nanoparticles were assigned using ImageJ software. A bivariate K-function analysis tested the null hypothesis that the two populations of gold particles are spatially independent of each other.

$$K_{biv}\left(r\right)={\left(n_{b}+n_{s}\right)}^{-1}\;\left[n_{b}K_{sb}\left(r\right)+n_{s}K_{bs}\left(r\right)\right]$$

$${K_{bs}\left(r\right)=A\;{\left(n_{b}\times n_{s}\right)}^{-1}\sum }_{i=1}^{nb}\;\sum_{j=1}^{ns}\;w_{ij}1\left(\|x_{i}{-x}_{j}\|\leq r\right)$$

$${K_{sb}\left(r\right)=A\;{\left(n_{b}\times n_{s}\right)}^{-1}\sum }_{i=1}^{ns}\;\sum_{j=1}^{nb}\;w_{ij}1\left(\|x_{i}{-x}_{j}\|\leq r\right)$$

$$L_{biv}\left(r\right)–r=\sqrt{\frac{K_{biv}(r)}{\pi }}-r$$

K_biv_(r) denotes a bivariate estimator and includes two individual bivariate K-functions: K_bs_(r) calculates how the larger 6 nm gold particles (b = big gold) are distributed around each 2 nm small gold particle (s = small gold) as a function of distance r in nanometers, and K_sb_(r) assesses how small gold particles are distributed around each large gold particle. The variables n_b_ and n_s_ represent the number of 6 nm large gold and 2 nm small gold particles within a PM area of A, respectively. Other parameters retain the same definitions as in the univariate analysis. L_biv_(r) – r is a linear transformation of K_biv_(r) and is normalized against the 95% CI. An L_biv_(r) – r value of 0 suggests spatial segregation between the two populations of gold particles, whereas an L_biv_(r) – r value above the 95% CI at a given r indicates statistically meaningful colocalization at that distance. To generate the summary statistic for the extent of coclustering, the area under the curve for each L_biv_(r) – r plot was calculated within a fixed range of 10 < r < 110 nm and was defined as bivariate L_biv_(r) – r integrated (LBI):

$${\text{LBI}=\int }_{10}^{110}Std\;L_{biv}\left(r\right)\;–\;r.\;dr$$

For each experimental condition, more than 15 apical PM sheets were imaged, analyzed, and pooled, and the results were presented as mean LBI values ± SD.

## Statistical analysis

All data are expressed as mean ± SD. Each “biological replicate” (*e.g.,* n number) used cells from a separate preparation of CMs from different animals. Statistical analyses were performed using Sigma Plot (v15.0; grafiti.com/sigmaplot-v16/) or Prism (GraphPad; www.graphpad.com) for ANOVA analysis followed by multiple comparison tests between groups or with Excel for comparison between two groups by Student’s paired and unpaired *t* tests. Statistical tests are indicated for each dataset in figure legends. Statistical significance was set as ∗*p* < 0.05, ∗∗*p* < 0.01, ∗∗∗*p* < 0.001, and ^#^*p* < 0.05, ^##^*p* < 0.01.

## Data availability

The proteins identified by BioID (SS > 0.5) and FLAG-MS (SS > 0.7), along with associated parameters, are provided in Tables S1 and S2. All data have been deposited as a complete submission to the MassIVE repository and is accessible at ftp://massive-ftp.ucsd.edu/v07/MSV000096100/. The accession is MSV000096100 and the ProteomeXchange accession is PXD056822.

## Supporting information

This article contains supporting information.

## Conflict of interest

The authors declare that they have no conflicts of interest with the contents of this article.

## Supporting information

Supplement Table 2

Supplement_BioID paper

## References

[1] De Jong K.A., Nikolaev V.O. (2021). Multifaceted remodelling of cAMP microdomains driven by different aetiologies of heart failure. FEBS J..

[2] Chen X., Piacentino V., Furukawa S., Goldman B., Margulies K.B., Houser S.R. (2002). L-type Ca2+ channel density and regulation are altered in failing human ventricular myocytes and recover after support with mechanical assist devices. Circ. Res..

[3] Periasamy M., Bhupathy P., Babu G.J. (2007). Regulation of sarcoplasmic reticulum Ca2+ ATPase pump expression and its relevance to cardiac muscle physiology and pathology. Cardiovasc. Res..

[4] Bers D.M. (2008). Calcium cycling and signaling in cardiac myocytes. Annu. Rev. Physiol..

[5] Hovey L., Gamal El-Din T.M., Catterall W.A. (2022). Convergent regulation of Ca(V)1.2 channels by direct phosphorylation and by the small GTPase RAD in the cardiac fight-or-flight response. Proc. Natl. Acad. Sci. U. S. A..

[6] Levitan B.M., Ahern B.M., Aloysius A., Brown L., Wen Y., Andres D.A. (2021). Rad-GTPase contributes to heart rate *via* L-type calcium channel regulation. J. Mol. Cell Cardiol..

[7] Subramaniam G., Schleicher K., Kovanich D., Zerio A., Folkmanaite M., Chao Y.C. (2023). Integrated proteomics unveils nuclear PDE3A2 as a regulator of Cardiac Myocyte Hypertrophy. Circ. Res..

[8] Folkmanaite M., Zaccolo M. (2023). Regulation of cardiac function by cAMP nanodomains. Biosci. Rep..

[9] Hammond H.K., Penny W.F., Traverse J.H., Henry T.D., Watkins M.W., Yancy C.W. (2016). Intracoronary gene transfer of adenylyl cyclase 6 in patients with heart failure: a randomized clinical trial. JAMA Cardiol..

[10] Tang T., Hammond H.K., Firth A., Yang Y., Gao M.H., Yuan J.X. (2011). Adenylyl cyclase 6 improves calcium uptake and left ventricular function in aged hearts. J. Am. Coll. Cardiol..

[11] Guellich A., Gao S., Hong C., Yan L., Wagner T.E., Dhar S.K. (2010). Effects of cardiac overexpression of type 6 adenylyl cyclase affects on the response to chronic pressure overload. Am. J. Physiol. Heart Circ. Physiol..

[12] Vatner S.F., Park M., Yan L., Lee G.J., Lai L., Iwatsubo K. (2013). Adenylyl cyclase type 5 in cardiac disease, metabolism, and aging. Am. J. Physiol. Heart Circ. Physiol..

[13] Maghsoudi S., Shuaib R., Van Bastelaere B., Dakshinamurti S. (2024). Adenylyl cyclase isoforms 5 and 6 in the cardiovascular system: complex regulation and divergent roles. Front. Pharmacol..

[14] Ostrom K.F., LaVigne J.E., Brust T.F., Seifert R., Dessauer C.W., Watts V.J. (2022). Physiological roles of mammalian transmembrane adenylyl cyclase isoforms. Physiol. Rev..

[15] Dessauer C.W., Watts V.J., Ostrom R.S., Conti M., Dove S., Seifert R. (2017). International union of basic and clinical pharmacology. CI. Structures and small molecule modulators of mammalian adenylyl cyclases. Pharmacol. Rev..

[16] Timofeyev V., Myers R.E., Kim H.J., Woltz R.L., Sirish P., Heiserman J.P. (2013). Adenylyl cyclase subtype-specific compartmentalization: differential regulation of L-type Ca2+ current in ventricular myocytes. Circ. Res..

[17] Nikolaev V.O., Moshkov A., Lyon A.R., Miragoli M., Novak P., Paur H. (2010). Beta2-adrenergic receptor redistribution in heart failure changes cAMP compartmentation. Science.

[18] Marsden A.N., Dessauer C.W. (2019). Nanometric targeting of type 9 adenylyl cyclase in heart. Biochem. Soc. Trans..

[19] Baldwin T.A., Li Y., Marsden A.N., Rinne S., Garza-Carbajal A., Schindler R.F.R. (2022). POPDC1 scaffolds a complex of adenylyl cyclase 9 and the potassium channel TREK-1 in heart. EMBO Rep..

[20] Baldwin T.A., Dessauer C.W. (2018). Function of adenylyl cyclase in heart: the AKAP connection. J. Cardiovasc. Dev. Dis..

[21] Roux K.J., Kim D.I., Raida M., Burke B. (2012). A promiscuous biotin ligase fusion protein identifies proximal and interacting proteins in mammalian cells. J. Cell Biol..

[22] Branon T.C., Bosch J.A., Sanchez A.D., Udeshi N.D., Svinkina T., Carr S.A. (2018). Efficient proximity labeling in living cells and organisms with TurboID. Nat. Biotechnol..

[23] Kim D.I., Birendra K.C., Zhu W., Motamedchaboki K., Doye V., Roux K.J. (2014). Probing nuclear pore complex architecture with proximity-dependent biotinylation. Proc. Natl. Acad. Sci. U. S. A..

[24] Hacker B.M., Tomlinson J.E., Wayman G.A., Sultana R., Chan G., Villacres E. (1998). Cloning, chromosomal mapping, and regulatory properties of the human type 9 adenylyl cyclase (ADCY9). Genomics.

[25] Baldwin T.A., Li Y., Brand C.S., Watts V.J., Dessauer C.W. (2019). Insights into the regulatory properties of Human adenylyl cyclase type 9. Mol. Pharmacol..

[26] Qi C., Sorrentino S., Medalia O., Korkhov V.M. (2019). The structure of a membrane adenylyl cyclase bound to an activated stimulatory G protein. Science.

[27] Antoni F.A. (2020). The chilling of adenylyl cyclase 9 and its translational potential. Cell Signal..

[28] Gilbert G., Demydenko K., Dries E., Puertas R.D., Jin X., Sipido K. (2020). Calcium signaling in cardiomyocyte function. Cold Spring Harb. Perspect. Biol..

[29] Li Y., Hof T., Baldwin T.A., Chen L., Kass R.S., Dessauer C.W. (2019). Regulation of IKs potassium current by isoproterenol in adult cardiomyocytes requires type 9 adenylyl cyclase. Cells.

[30] Ackerman M.J., Mohler P.J. (2010). Defining a new paradigm for human arrhythmia syndromes: phenotypic manifestations of gene mutations in ion channel- and transporter-associated proteins. Circ. Res..

[31] Lazar A.M., Irannejad R., Baldwin T.A., Sundaram A.B., Gutkind J.S., Inoue A. (2020). G protein-regulated endocytic trafficking of adenylyl cyclase type 9. Elife.

[32] Ripoll L., Li Y., Dessauer C.W., von Zastrow M. (2024). Spatial organization of adenylyl cyclase and its impact on dopamine signaling in neurons. Nat. Commun..

[33] Cui L., Li H., Xi Y., Hu Q., Liu H., Fan J. (2022). Vesicle trafficking and vesicle fusion: mechanisms, biological functions, and their implications for potential disease therapy. Mol. Biomed..

[34] Yen Y.C., Li Y., Chen C.L., Klose T., Watts V.J., Dessauer C.W. (2024). Structure of adenylyl cyclase 5 in complex with Gbetagamma offers insights into ADCY5-related dyskinesia. Nat. Struct. Mol. Biol..

[35] Willoughby D., Cooper D.M. (2007). Organization and Ca2+ regulation of adenylyl cyclases in cAMP microdomains. Physiol. Rev..

[36] Sadana R., Dessauer C.W. (2009). Physiological roles for G protein-regulated adenylyl cyclase isoforms: insights from knockout and overexpression studies. Neurosignals.

[37] Okumura S., Kawabe J., Yatani A., Takagi G., Lee M.C., Hong C. (2003). Type 5 adenylyl cyclase disruption alters not only sympathetic but also parasympathetic and calcium-mediated cardiac regulation. Circ. Res..

[38] Tang T., Gao M.H., Lai N.C., Firth A.L., Takahashi T., Guo T. (2008). Adenylyl cyclase type 6 deletion decreases left ventricular function *via* impaired calcium handling. Circulation.

[39] Okumura S., Takagi G., Kawabe J., Yang G., Lee M.C., Hong C. (2003). Disruption of type 5 adenylyl cyclase gene preserves cardiac function against pressure overload. Proc. Natl. Acad. Sci. U. S. A..

[40] Okumura S., Vatner D.E., Kurotani R., Bai Y., Gao S., Yuan Z. (2007). Disruption of type 5 adenylyl cyclase enhances desensitization of cyclic adenosine monophosphate signal and increases Akt signal with chronic catecholamine stress. Circulation.

[41] Yan L., Vatner D.E., O'Connor J.P., Ivessa A., Ge H., Chen W. (2007). Type 5 adenylyl cyclase disruption increases longevity and protects against stress. Cell.

[42] Nakamura M., Sadoshima J. (2018). Mechanisms of physiological and pathological cardiac hypertrophy. Nat. Rev. Cardiol..

[43] Barbier A.J., Poppleton H.M., Yigzaw Y., Mullenix J.B., Wiepz G.J., Bertics P.J. (1999). Transmodulation of epidermal growth factor receptor function by cyclic AMP-dependent protein kinase. J. Biol. Chem..

[44] Liebmann C. (2001). Regulation of MAP kinase activity by peptide receptor signalling pathway: paradigms of multiplicity. Cell Signal..

[45] Caldwell G.B., Howe A.K., Nickl C.K., Dostmann W.R., Ballif B.A., Deming P.B. (2012). Direct modulation of the protein kinase A catalytic subunit α by growth factor receptor tyrosine kinases. J. Cell Biochem..

[46] Deckx S., Johnson D.M., Rienks M., Carai P., Van Deel E., Van der Velden J. (2019). Extracellular SPARC increases cardiomyocyte contraction during health and disease. PLoS One.

[47] Lu B., Yu H., Zwartbol M., Ruifrok W.P., van Gilst W.H., de Boer R.A. (2012). Identification of hypertrophy- and heart failure-associated genes by combining *in vitro* and *in vivo* models. Physiol. Genomics.

[48] Schmitz B., De Maria R., Gatsios D., Chrysanthakopoulou T., Landolina M., Gasparini M. (2014). Identification of genetic markers for treatment success in heart failure patients. Circ. Cardiovasc. Genet..

[49] Li Y., Baldwin T.A., Wang Y., Subramaniam J., Carbajal A.G., Brand C.S. (2017). Loss of type 9 adenylyl cyclase triggers reduced phosphorylation of Hsp20 and diastolic dysfunction. Sci. Rep..

[50] Gavillet B., Rougier J.-S., Domenighetti A.A., Behar R., Boixel C., Ruchat P. (2006). Cardiac sodium channel Nav1.5 is regulated by a Multiprotein complex composed of syntrophins and dystrophin. Circ. Res..

[51] Iqbal S.M., Lemmens-Gruber R. (2019). Phosphorylation of cardiac voltage-gated sodium channel: potential players with multiple dimensions. Acta Physiol. (Oxf).

[52] Tang W.J., Stanzel M., Gilman A.G. (1995). Truncation and alanine-scanning mutants of type I adenylyl cyclase. Biochemistry.

[53] Gu C., Cali J.J., Cooper D.M. (2002). Dimerization of mammalian adenylate cyclases. Eur. J. Biochem..

[54] Chen-Goodspeed M., Lukan A.N., Dessauer C.W. (2005). Modeling of G alpha(s) and G alpha(i) regulation of human type V and VI adenylyl cyclase. J. Biol. Chem..

[55] Khanppnavar B., Schuster D., Lavriha P., Uliana F., Ozel M., Mehta V. (2024). Regulatory sites of CaM-sensitive adenylyl cyclase AC8 revealed by cryo-EM and structural proteomics. EMBO Rep..

[56] Baragli A., Grieco M.L., Trieu P., Villeneuve L.R., Hebert T.E. (2008). Heterodimers of adenylyl cyclases 2 and 5 show enhanced functional responses in the presence of Galpha s. Cell Signal..

[57] Irannejad R., Tsvetanova N.G., Lobingier B.T., von Zastrow M. (2015). Effects of endocytosis on receptor-mediated signaling. Curr. Opin. Cell Biol..

[58] Irannejad R., Tomshine J.C., Tomshine J.R., Chevalier M., Mahoney J.P., Steyaert J. (2013). Conformational biosensors reveal GPCR signalling from endosomes. Nature.

[59] Vilardaga J.-P., Jean-Alphonse F.G., Gardella T.J. (2014). Endosomal generation of cAMP in GPCR signaling. Nat. Chem. Biol..

[60] Irannejad R., Pessino V., Mika D., Huang B., Wedegaertner P.B., Conti M. (2017). Functional selectivity of GPCR-directed drug action through location bias. Nat. Chem. Biol..

[61] Lin T.Y., Mai Q.N., Zhang H., Wilson E., Chien H.C., Yee S.W. (2023). Cardiac contraction and relaxation are regulated by distinct subcellular cAMP pools. Nat. Chem. Biol..

[62] Nash C.A., Wei W., Irannejad R., Smrcka A.V. (2019). Golgi localized beta1-adrenergic receptors stimulate Golgi PI4P hydrolysis by PLCepsilon to regulate cardiac hypertrophy. Elife.

[63] Turcotte M.G., Samuelsson A.-M., Possidento S.M., Li J., Qin Z., Kapiloff M.S. (2024). The unique role of intracellular perinuclear β-Adrenergic receptors in defining signaling compartmentation and pathological cardiac remodeling. bioRxiv.

[64] Cheng H., Farquhar M.G. (1976). Presence of adenylate cyclase activity in Golgi and other fractions from rat liver. II. Cytochemical localization within Golgi and ER membranes. J. Cell Biol..

[65] Nassari S., Del Olmo T., Jean S. (2020). Rabs in signaling and embryonic development. Int. J. Mol. Sci..

[66] Hager H.A., Roberts R.J., Cross E.E., Proux-Gillardeaux V., Bader D.M. (2010). Identification of a novel bves function: regulation of vesicular transport. EMBO J..

[67] Caceres P.S., Mendez M., Ortiz P.A. (2014). Vesicle-associated membrane protein 2 (VAMP2) but not VAMP3 mediates cAMP-stimulated trafficking of the renal Na+-K+-2Cl- co-transporter NKCC2 in thick ascending limbs. J. Biol. Chem..

[68] Bakr M., Jullié D., Krapivkina J., Paget-Blanc V., Bouit L., Petersen J.D. (2021). The vSNAREs VAMP2 and VAMP4 control recycling and intracellular sorting of post-synaptic receptors in neuronal dendrites. Cell Rep..

[69] Gouraud S., Laera A., Calamita G., Carmosino M., Procino G., Rossetto O. (2002). Functional involvement of VAMP/synaptobrevin-2 in cAMP-stimulated aquaporin 2 translocation in renal collecting duct cells. J. Cell Sci..

[70] Vuolo L., Herrera A., Torroba B., Menendez A., Pons S. (2015). Ciliary adenylyl cyclases control the Hedgehog pathway. J. Cell Sci..

[71] Somatilaka B.N., Hwang S.H., Palicharla V.R., White K.A., Badgandi H., Shelton J.M. (2020). Ankmy2 prevents smoothened-independent hyperactivation of the hedgehog pathway *via* cilia-Regulated adenylyl cyclase signaling. Dev. Cell.

[72] Anvarian Z., Mykytyn K., Mukhopadhyay S., Pedersen L.B., Christensen S.T. (2019). Cellular signalling by primary cilia in development, organ function and disease. Nat. Rev. Nephrol..

[73] Piggott L.A., Bauman A.L., Scott J.D., Dessauer C.W. (2008). The A-kinase anchoring protein Yotiao binds and regulates adenylyl cyclase in brain. Proc. Natl. Acad. Sci. U. S. A..

[74] Kapiloff M.S., Piggott L.A., Sadana R., Li J., Heredia L.A., Henson E. (2009). An adenylyl cyclase-mAKAPbeta signaling complex regulates cAMP levels in cardiac myocytes. J. Biol. Chem..

[75] Xie K., Masuho I., Shih C.C., Cao Y., Sasaki K., Lai C.W. (2015). Stable G protein-effector complexes in striatal neurons: mechanism of assembly and role in neurotransmitter signaling. Elife.

[76] Cormier K.W., Larsen B., Gingras A.C., Woodgett J.R. (2023). Interactomes of glycogen synthase Kinase-3 isoforms. J. Proteome Res..

[77] Shteynberg D., Deutsch E.W., Lam H., Eng J.K., Sun Z., Tasman N. (2011). iProphet: Multi-level integrative analysis of shotgun proteomic data improves peptide and protein identification rates and error estimates. Mol. Cell Proteomics.

[78] Teo G., Liu G., Zhang J., Nesvizhskii A.I., Gingras A.C., Choi H. (2014). SAINTexpress: improvements and additional features in Significance Analysis of INTeractome software. J. Proteomics.

[79] Kolberg L., Raudvere U., Kuzmin I., Adler P., Vilo J., Peterson H. (2023). g:Profiler—interoperable web service for functional enrichment analysis and gene identifier mapping (2023 update). Nucleic Acids Res..

[80] Ashburner M., Ball C.A., Blake J.A., Botstein D., Butler H., Cherry J.M. (2000). Gene ontology: tool for the unification of biology. The gene ontology Consortium. Nat. Genet..

[81] Thomas P.D., Ebert D., Muruganujan A., Mushayahama T., Albou L.P., Mi H. (2022). PANTHER: making genome-scale phylogenetics accessible to all. Protein Sci..

[82] Orre L.M., Vesterlund M., Pan Y., Arslan T., Zhu Y., Fernandez Woodbridge A. (2019). SubCellBarCode: proteome-wide mapping of protein localization and relocalization. Mol. Cell.

[83] Consortium T.U. (2022). UniProt: the Universal protein knowledgebase in 2023. Nucleic Acids Res..

[84] Dessauer C.W. (2002). Kinetic analysis of the action of P-site analogs. Methods Enzymol..

[85] Prior I.A., Parton R.G., Hancock J.F. (2003). Observing cell surface signaling domains using electron microscopy. Sci. STKE.

[86] Zhou Y., Hancock J.F. (2021). Super-Resolution imaging and spatial analysis of RAS on intact plasma membrane sheets. Methods Mol. Biol..

[87] Arora N., Mu H., Liang H., Zhao W., Zhou Y. (2024). RAS G-domains allosterically contribute to the recognition of lipid headgroups and acyl chains. J. Cell Biol..

