# Supplementary material for: Proximity-dependent proteomics and network analysis of adenylyl cyclase isoforms 5, 6, and 9 in cardiomyocytes
Source: J Biol Chem. 2025 Jul 31;301(9):110539. doi: 10.1016/j.jbc.2025.110539 (PMC12446771; doi:10.1016/j.jbc.2025.110539)
Supplement: Supplement_BioID paper [file mmc3.pdf]

Supporting Information

Proximity-Dependent Proteomics and Network Analysis of Adenylyl Cyclase Isoforms 5, 6, and 9 in Cardiomyocytes

Taeyeop Park[1], Yong Li[1], Neha Arora[1], Anibal Garza-Carbajal[1], Karen Colwill[2], Cassandra J. Wong[2], Yong Zhou[1] and Carmen W. Dessauer[1#]

[1]Department of Integrative Biology and Pharmacology, McGovern Medical School at the University of Texas Health Science Center, Houston TX; [2]Lunenfeld-Tanenbaum Research Institute, Sinai Health, Toronto, Ontario

[#]To whom correspondence may be addressed: Carmen.W.Dessauer@uth.tmc.edu

Figure S1

Figure S2

Figure S3

Figure S4

Figure S5

Figure S6

Figure S7

Table S1 – Excel file

Table S2 – Excel file

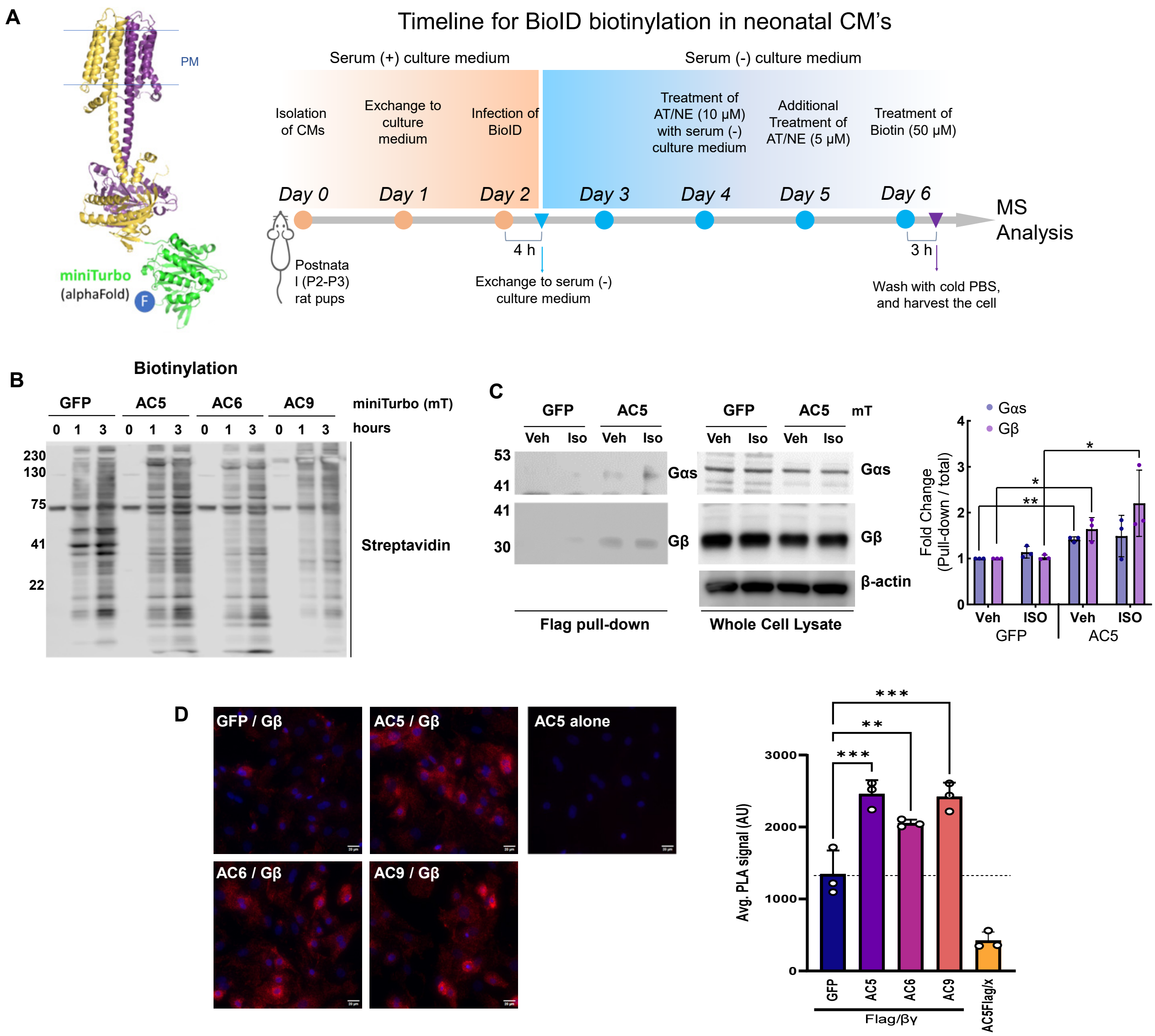

Figure S1. Optimization of biotinylation and validation of BioID constructs in neonatal CMs. *A*, ACx-miniTurbo-FLAG structural model and experimental timeline for preparation of samples for MS analysis. *B*, Time course of biotin-labeling. CMs were treated for the indicated times with 50 μM biotin and proteins resolved by SDS-PAGE. Biotin-labeled proteins were detected by Western blot using streptavidin-HRP and a representative image is shown (n=3). *C*, Validation of AC5-mT construct. Following CMs infection with AC5-mT versus GFP-mT for 2 days, cells were treated with ISO (10 nM) for 2 min. Interaction of Gαs and Gβγ with AC5 was assessed by FLAG immunoprecipitation and western blot analysis. A representative image and quantification of interaction of endogenous Gαs and Gβγ with AC5 is shown (n=3, mean ± S.D., *p<0.05; ** p<0.01, compared to GFP-mT controls.). *D,* Proximity ligation assay (PLA) analysis confirms Gβγ interaction with ACs in CMs. CMs were infected with indicated AC-mT and GFP-mT for 2 days. PLA was performed using anti-FLAG and anti-Gβ antibodies. PLA signal was undetectable in negative control experiment, where only FLAG antibody was incubated with the PLA probes. Quantification of AC-Gβ PLA and representative images are shown. mean ± S.D., n=3, ** p<0.01; *** p<0.001, one-way ANOVA compared to GFP-mT control using Dunnett's multiple comparisons test.

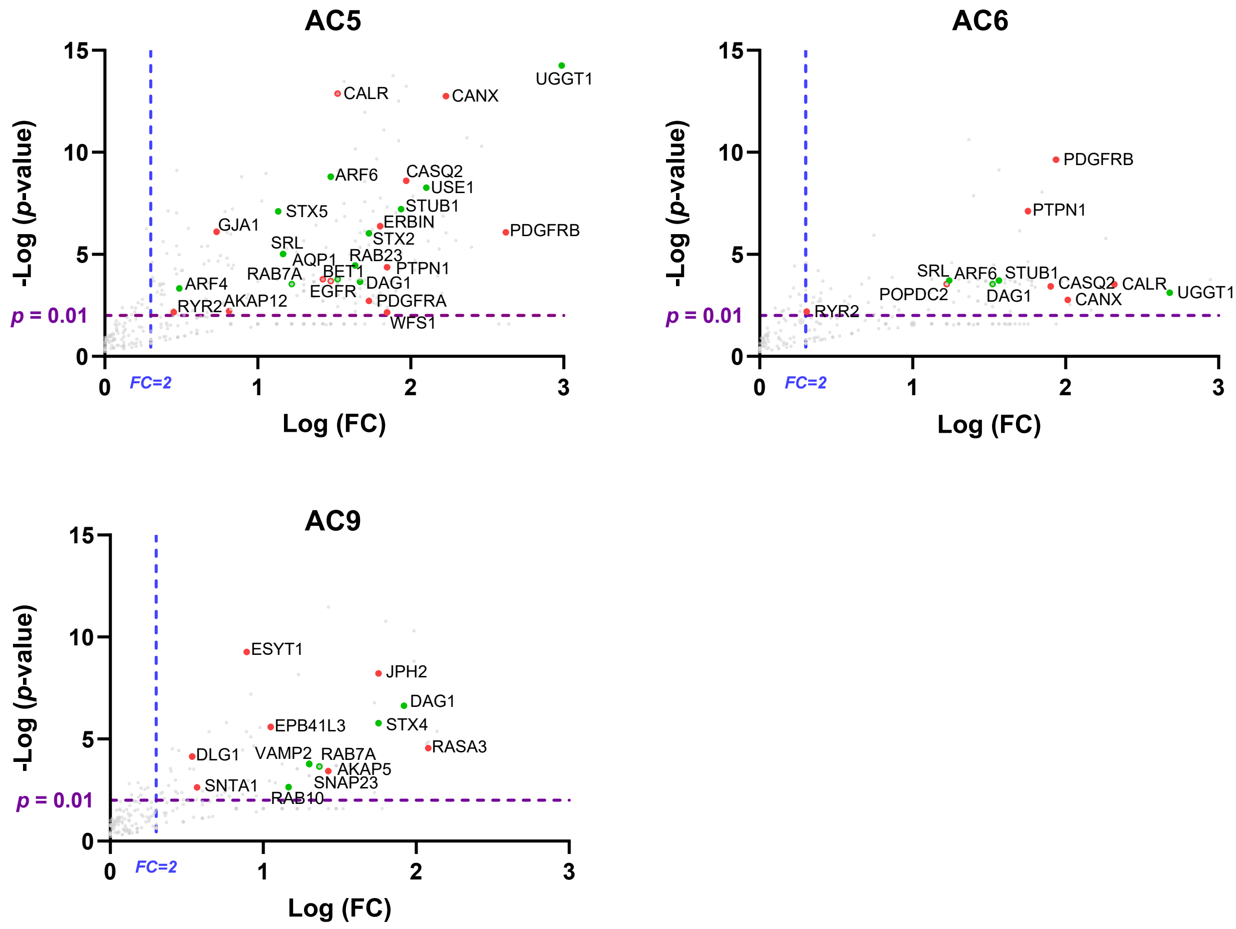

Figure S2. Volcano plots of proximity proteomics for ACx-mT compared to GFP-mT and non-infected controls. The $\log_{10}$(fold change) was plotted versus $-\log_{10}$(p-value), with the p=0.01 and fold change (FC)=2 indicated by dashed lines. The p-value was calculated using paired student t-test for all vehicle treated samples (n=3) against all controls. NE-treated samples with SS>0.7 that do not appear in vehicle datasets are also included. AC near neighbor proteins corresponding to protein trafficking (green) and cardiac function / PM organization (red) are labeled as described in Figure 6.

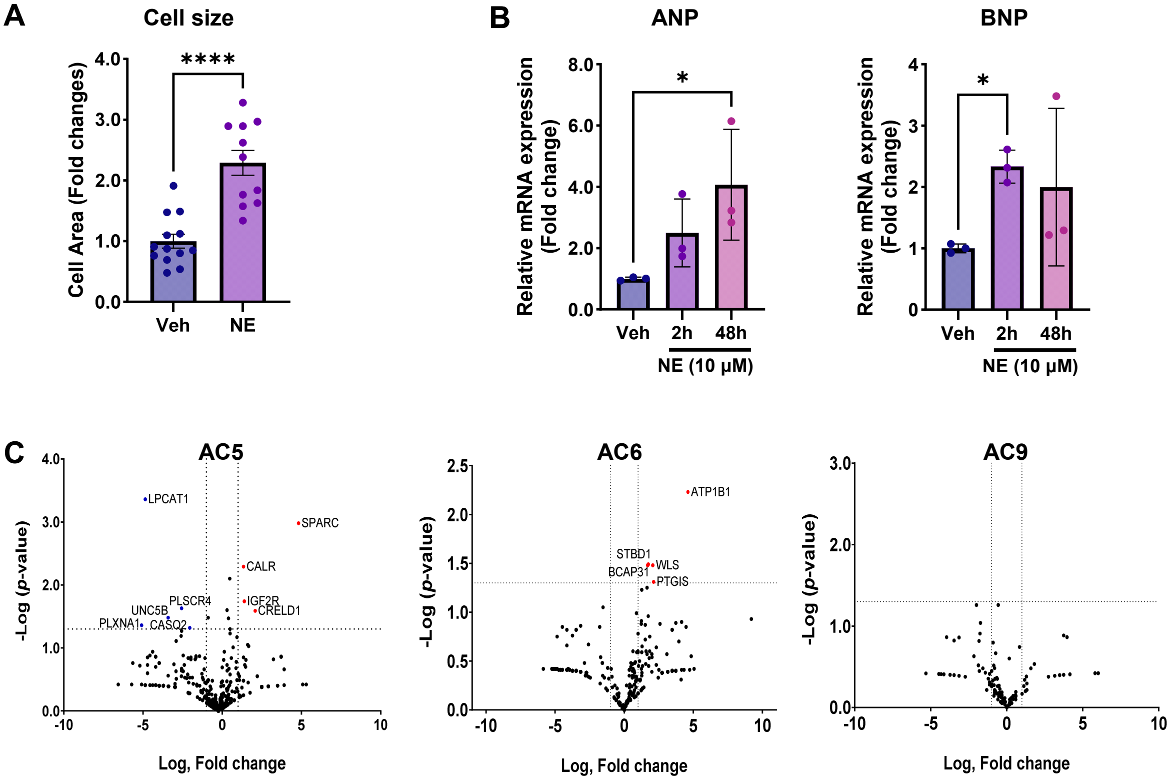

Figure S3. Alterations in BioID datasets upon CM hypertrophy. *A*, Hypertrophy assessment of CMs treated with norepinephrine (NE). Cell size was measured by ImageJ (v1.52p) using α-actinin staining. *B*. Expression of atrial natriuretic peptide (ANP) and B-type natriuretic peptide (BNP) mRNA was quantified using qPCR and normalized to GAPDH (n=3, one-way ANOVA, Dunett's multiple comparisons test compared to Veh). *C*. Volcano plots illustrating differential AC near neighbors upon NE treatment versus control. The $-\log_{10}$ *p*-value (y-axis) is plotted against the log fold change (x-axis) in NE versus vehicle treatment groups. Each dot represents a single protein, with those significantly increased and decreased colored red and blue, respectively. The horizontal dashed line corresponds to $p = 0.05$; vertical lines correspond to ± 2-fold change.

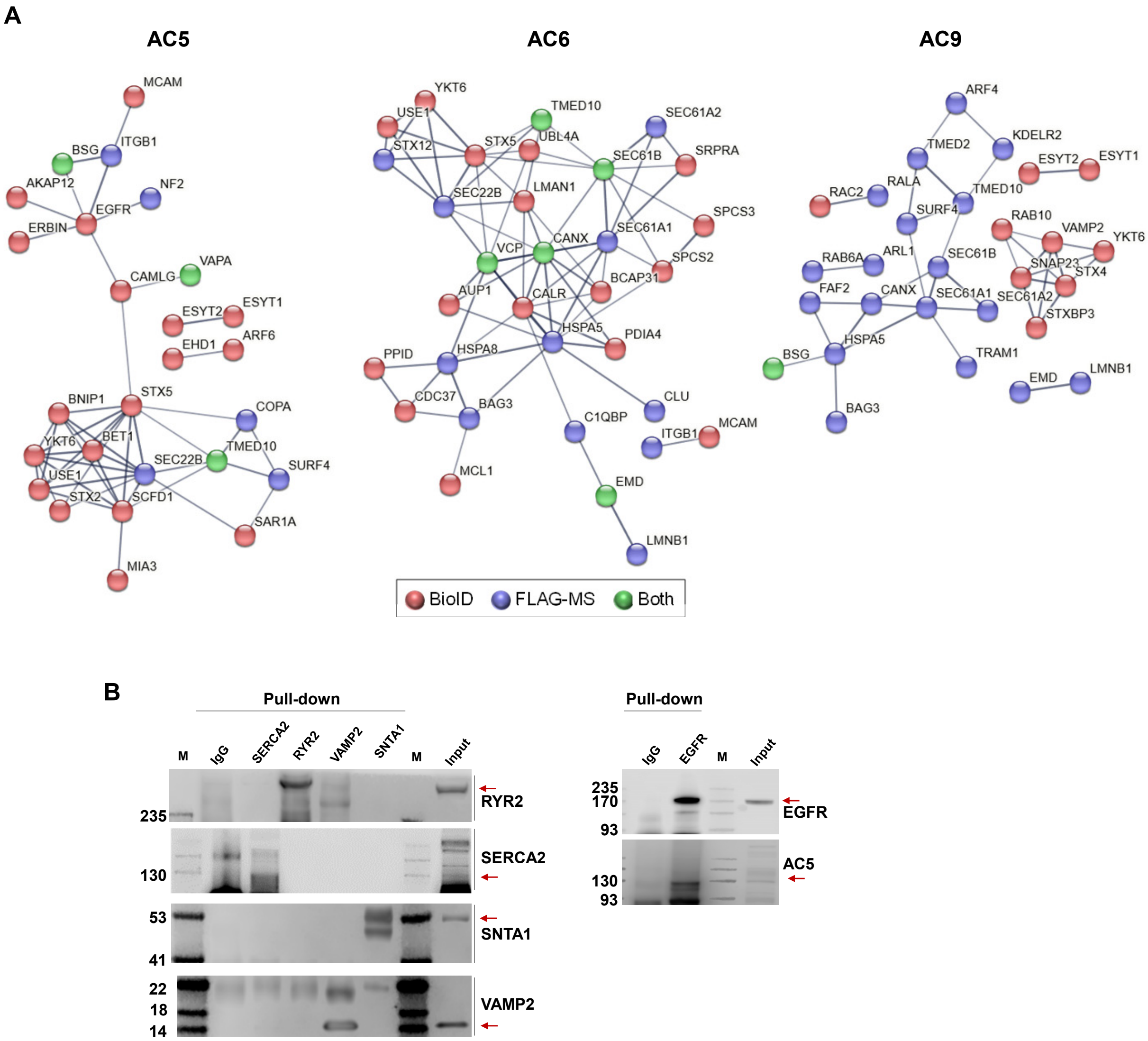

Figure S4. Validation of BioID and FLAG-MS datasets. *A*, STRING analysis of BioID and FLAG-MS datasets for proteins associated with protein transport (GO:0015031) terms from BioID (SS ≥ 0.7, red) and FLAG-MS (SS ≥ 0.9, blue) were clustered by STRING (Ver 12.0. high confidence=0.9). Protein circles are colored according to those detected by BioID alone (red), by FLAG-MS alone (blue), or both (green circles). *B*, Western blot control for endogenous IP-AC assay. A portion of the lysate and pull-down from figure 5*C* were subjected to Western blotting using the indicated antibodies, as evidence of their specificity. A representative image is shown, n=3. Right panel, evidence of co-immunoprecipitation of endogenous AC5 and EGFR from neonatal cardiomyocytes.

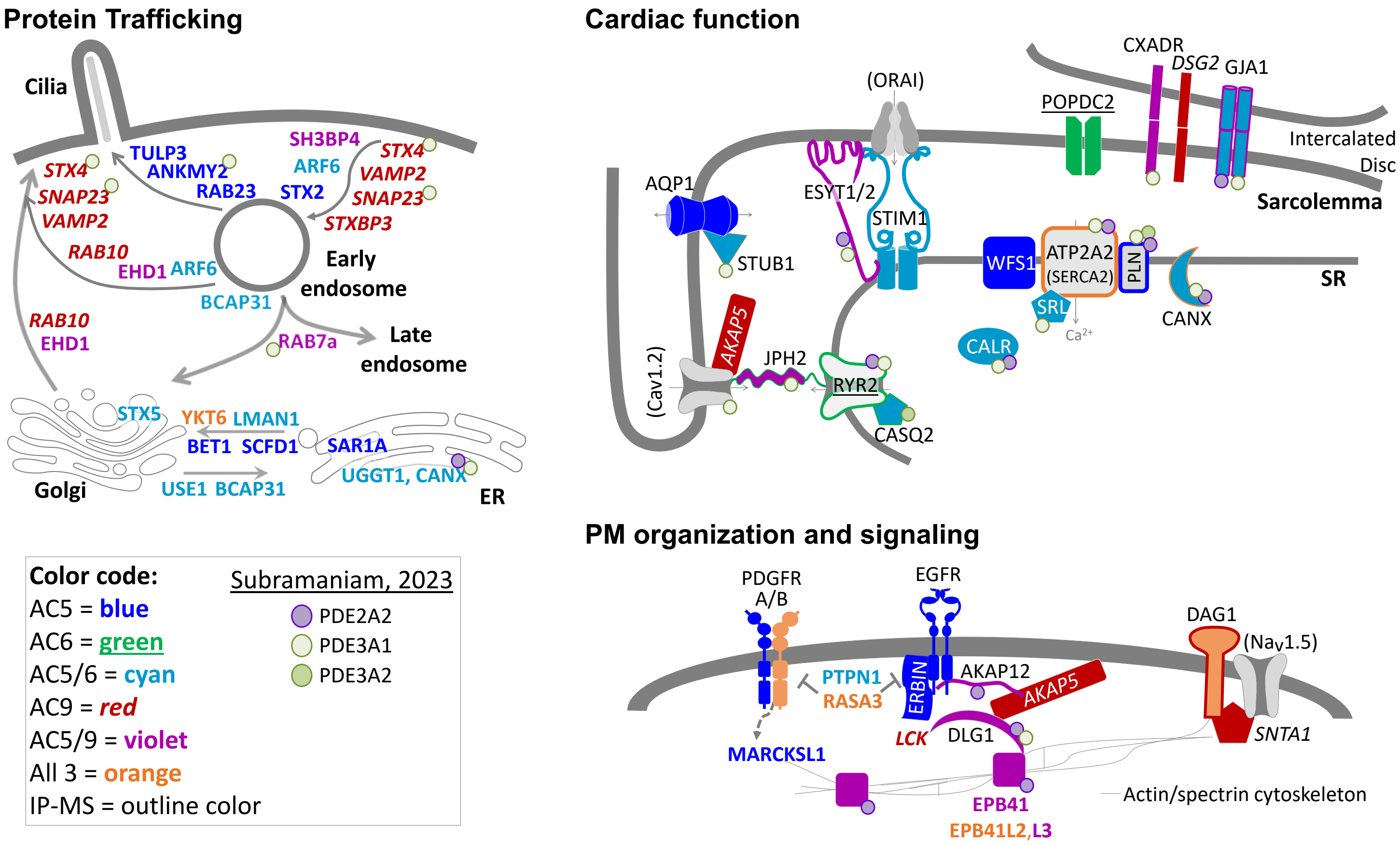

Figure S5. Comparison of AC and PDE proximal interactomes in cardiomyocytes. The schematic is identical to that of Fig 6, showing the AC complex map, except that the PDE interactome based on Subramaniam et al (7) is added to the figure. The proteins identified as AC proximal complexes are color coded with respect to the individual AC isoform (see legend for details). Shape outline colors represent FLAG-MS identified proteins; those filled grey (i.e. RYR2 and ATP2A2) were identified by FLAG-MS alone. Proteins also identified in PDE databases are shown associated with green or purple circles for PDE2 and PDE3, respectively.

A

BioID (Veh/NE combined)

	GFP	AC5	AC6	AC9
**Adcy5**	0		16	4
**Adcy6**	0	11		0
**Adcy9**	0	0	0	

FLAG-MS

	GFP	AC5	AC6	AC9
**Adcy5**	0		6	8
**Adcy6**	0	6		0
**Adcy9**	4	1	0	

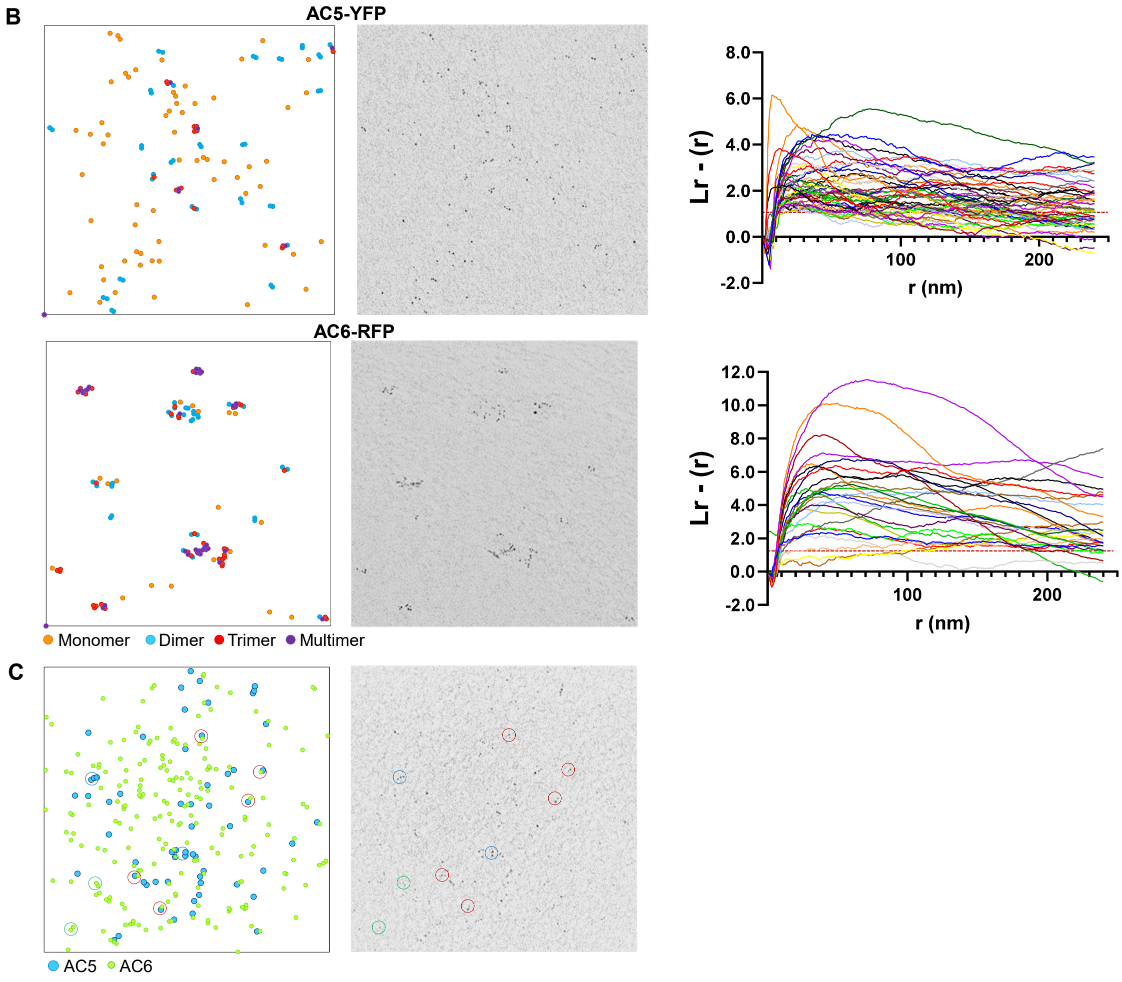

Figure S6. Heterodimerization of AC5:AC6. *A*, BioID and FLAG-MS evidence for AC5:AC6 heterodimerization. The number of unique peptides for AC5, AC6, and AC9 detected by BioID and FLAG-MS are shown. This is likely an under-representation, as AC5 and AC6 show high homology and both BioID and FLAG-MS detected many shared peptides for AC5 and AC6. *B*, Intact PM sheets from BHK cells were attached to EM grids and immunolabeled with anti-YFP (AC5) or anti-RFP (AC6) antibody conjugated to gold particles (4.5 nm) and imaged using transmission EM at 100,000× magnification. A sample electron micrograph and superimposed spatial clustering distribution of an intact PM sheet with an area of 1 μm^2 is shown for AC5-YFP (top) and AC6-RFP (bottom). The graphs (right) show the extent of nanoclustering, where individual $L(r) - r$ was plotted versus r. $L(r) - r$ values above the 99% confidence interval of 1.0 indicate statistically meaningful clustering. *C*, Bivariate co-clustering analysis characterizes the co-localization between AC5-YFP and AC6-RFP. A sample electron micrograph and superimposed spatial clustering distribution of an intact PM sheet (1 μm^2) for BHK cells expressing both AC5-YFP and AC6-RFP. Gold particles were conjugated to anti-YFP antibody (6 nm, AC5, blue) and to anti-RFP antibody (2 nm, AC6, green). Examples of heterodimers are indicated with red circles, while homodimers or homomultimers of AC5 and AC6 are indicated with blue and green circles, respectively.

Uncropped blots, Fig 5C

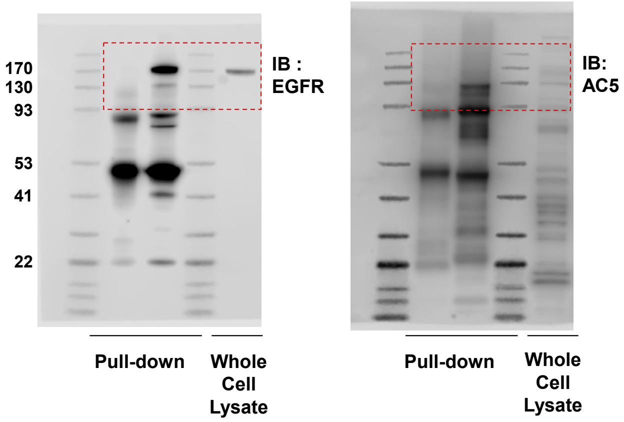

Uncropped blots, S1C

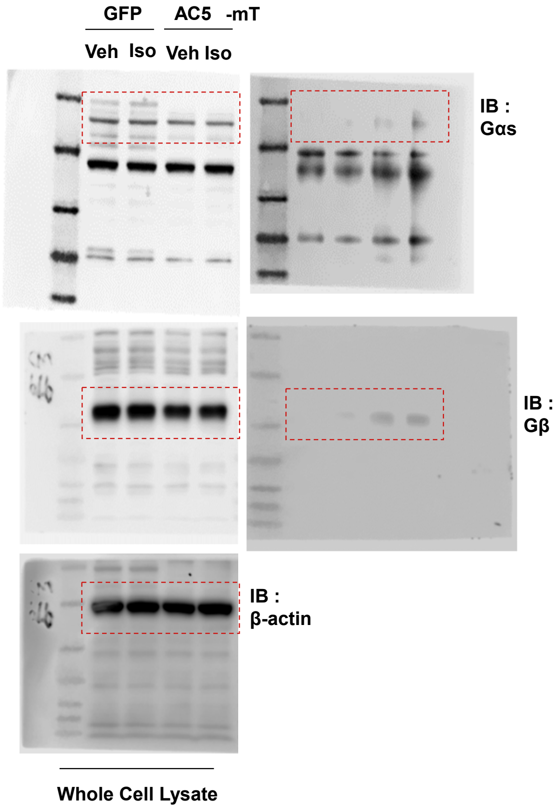

Uncropped blots, S3B

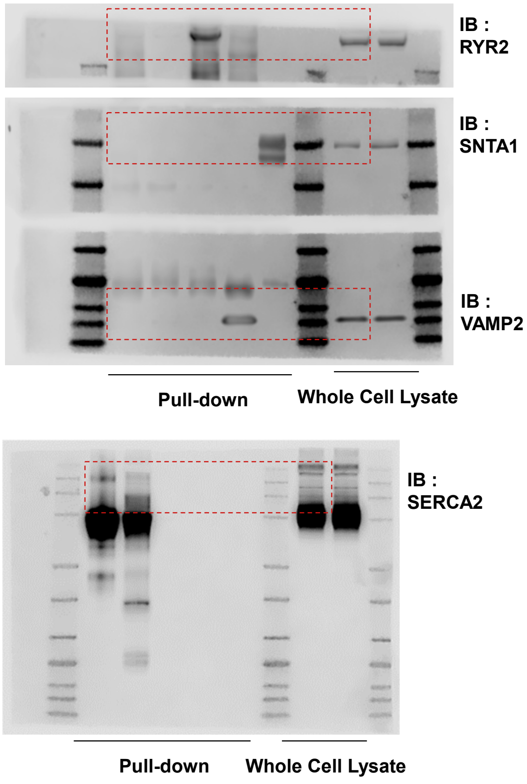

Figure S7. Uncropped images of western blot
